# Ancient DNA Reveals That the Genetic Structure of the Northern Han Chinese Was Shaped Prior to 3,000 Years Ago

**DOI:** 10.1371/journal.pone.0125676

**Published:** 2015-05-04

**Authors:** Yong-Bin Zhao, Ye Zhang, Quan-Chao Zhang, Hong-Jie Li, Ying-Qiu Cui, Zhi Xu, Li Jin, Hui Zhou, Hong Zhu

**Affiliations:** 1 College of Life Science, Jilin University, Changchun, China; 2 College of Life Science, Jilin Normal University, Siping, China; 3 Laboratory of Ancient DNA, Research Center for Chinese Frontier Archaeology of Jilin University, Changchun, China; 4 Ministry of Education (MOE) Key Laboratory of Contemporary Anthropology and Center for Evolutionary Biology, School of Life Sciences and Institutes of Biomedical Sciences, Fudan University, Shanghai, China; University of York, UNITED KINGDOM

## Abstract

The Han Chinese are the largest ethnic group in the world, and their origins, development, and expansion are complex. Many genetic studies have shown that Han Chinese can be divided into two distinct groups: northern Han Chinese and southern Han Chinese. The genetic history of the southern Han Chinese has been well studied. However, the genetic history of the northern Han Chinese is still obscure. In order to gain insight into the genetic history of the northern Han Chinese, 89 human remains were sampled from the Hengbei site which is located in the Central Plain and dates back to a key transitional period during the rise of the Han Chinese (approximately 3,000 years ago). We used 64 authentic mtDNA data obtained in this study, 27 Y chromosome SNP data profiles from previously studied Hengbei samples, and genetic datasets of the current Chinese populations and two ancient northern Chinese populations to analyze the relationship between the ancient people of Hengbei and present-day northern Han Chinese. We used a wide range of population genetic analyses, including principal component analyses, shared mtDNA haplotype analyses, and geographic mapping of maternal genetic distances. The results show that the ancient people of Hengbei bore a strong genetic resemblance to present-day northern Han Chinese and were genetically distinct from other present-day Chinese populations and two ancient populations. These findings suggest that the genetic structure of northern Han Chinese was already shaped 3,000 years ago in the Central Plain area.

## Introduction

**Fig 1:**
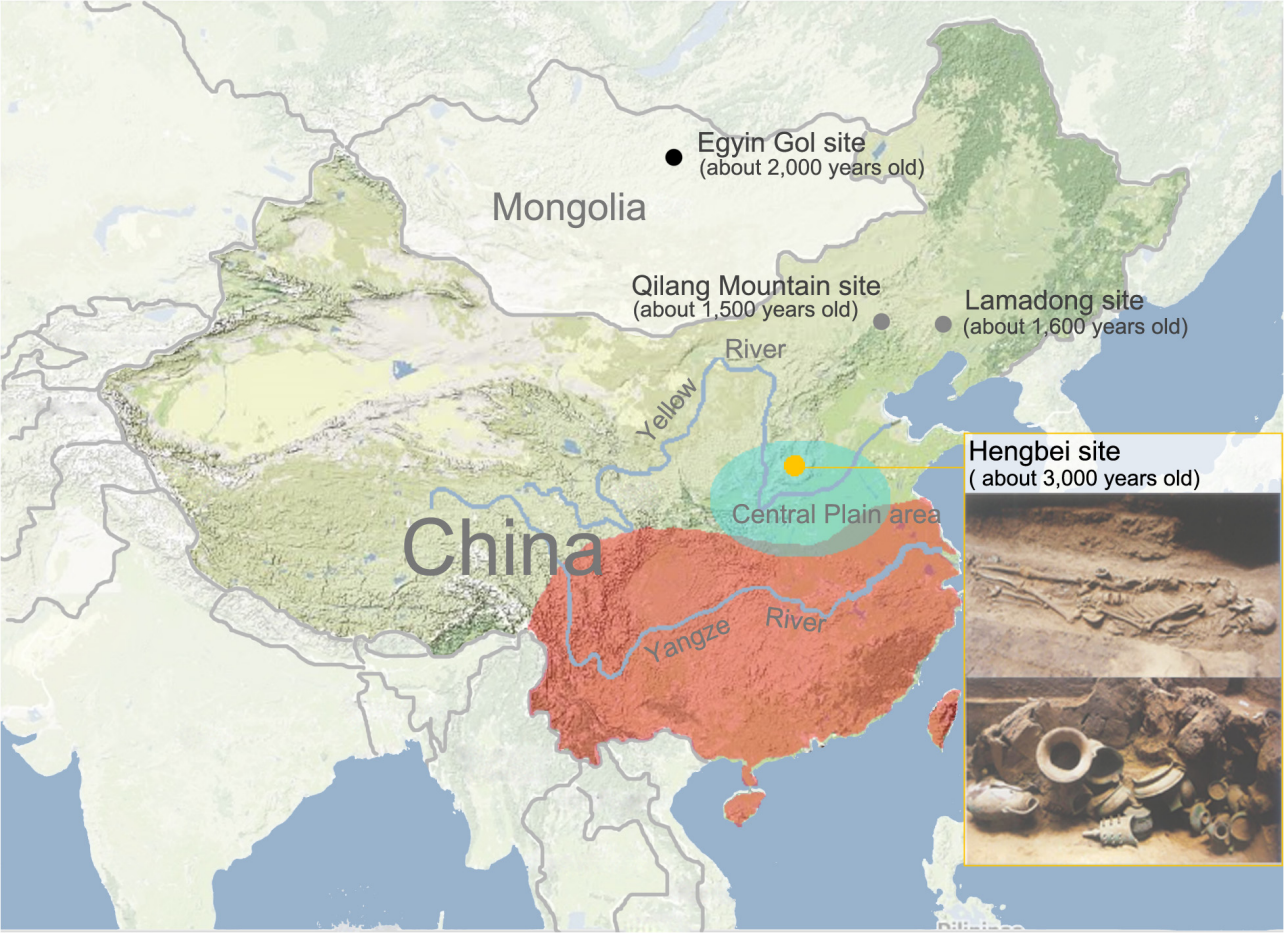
Geographic location of the Hengbei (HB) and Central Plain regions. HB is marked by a yellow circle, and the Central Plain region is indicated by a cyan ellipse. XN population is marked by a black circle, and XB populations are marked by two grey circles. The red area marked on the map represents the location of the southern Han, and the green area marked in China represents the location of the northern Han. The human remains and sacrificial vessels in the two photos in the lower right corner were both excavated from the Hengbei site.

The Han Chinese are the largest ethnic group in the world and have a current population of a staggering 1.3 billion individuals [1]. According to historical documents, the generally accepted view is that the Han Chinese can trace their origins to the Huaxia ethnic group, which formed during the Shang and Zhou dynasties (21st–8th centuries BC) in the Central Plain region of China (Fig 1) [2]. During the Han Dynasty (260 BC-220 AD), the Huaxia ethnic group developed into a tribe known as the Han Chinese [3]. Because of their advanced agriculture and technology, this group migrated northward into regions inhabited by many ancient northern ethnic groups that were most likely Altaic in origin [4]. In addition, they migrated south into regions originally inhabited by ancient southern ethnic groups, including those speaking the Daic, Austro-Asiatic, and Hmong-Mien languages [3]. Historically, the Han Chinese dispersed across China, becoming the largest of the 56 officially recognized ethnic groups.

Genetic studies can substantially improve our ability to understand the origins of human populations, including where and when they migrated [5–8]. Geneticists are increasingly paying close attention to the Han Chinese, studying the group’s origin and development. To date, studies of classic genetic markers and microsatellites have revealed that the Han Chinese can be divided into two distinct groups: the northern Han Chinese (NH) and the southern Han Chinese (SH) [9,10]. Based on present-day genetic data from NH, SH, and southern minorities, the genetic history of the SH group has been well studied. The consensus is that the Han Chinese migrated south and contributed greatly to the paternal gene pool of the SH, whereas the Han Chinese and ancient southern ethnic groups both contributed almost equally to the SH maternal gene pool [11]. However, the genetic history of the NH is still obscure. Currently, NH populations inhabit much of northern China, including the Central Plain and many outer regions that were inhabited by ancient northern ethnic groups (Fig 1). The Han Chinese or their ancestors who migrated northward from the Central Plain might have mixed with ancient northern ethnic groups or culturally assimilated the native population. This scenario would indicate that the Han Chinese living in different areas should have genetic profiles that differ from each other. However, genetic analyses have shown that there are no significant differences among the northern Han Chinese populations [12], which has led to conflicting arguments on whether the genetic structure of the NH is the result of an earlier ethnogenesis or, instead, results from a combination of population admixture and continuous migration of the Han Chinese. The addition of ancient DNA analysis on ancient Han Chinese samples provides increased information that can be used to reconstruct recent human evolutionary events in ancient China [13].

Until now, only a few genetic studies have investigated the ancient Han Chinese or their ancestors. These studies have been restricted by small sample sizes [14,15], high levels of kinship among samples [16], and short fragments of mitochondrial DNA (mtDNA) [17,18] and thus provide limited insights into the genetic history of the Han Chinese. Recently, a large number of graves were excavated at a necropolis called Hengbei located in the southern part of Shanxi Province, China, on the Central Plain (Fig 1), that dates back to approximately 3,000 years ago (Zhou dynasty) [19], a key transitional period for the rise of the Han Chinese. In a previous study investigating when haplogroup Q1a1 entered the genetic pool of the Han Chinese, we analyzed Y chromosome single nucleotide polymorphisms (SNPs) from human remains excavated from the Hengbei (HB) site and identified haplogroups for 27 samples[20]. In the present study, we attempted to extract DNA from 89 human remains. Using a combination of Y chromosome SNPs and mtDNA genetic data, we uncover aspects of the genetic structure of the ancient people from the Central Plain region and begin to determine the genetic legacy of the northern Han Chinese in both the maternal and paternal lineages.

## Materials and Methods

### Site and samples

The Hengbei site (35°29’N and 111°27’S) contains a large number (~1,300) of graves and is located in Jiang County, Shanxi Province, China, an area that was part of the suburbs of the capital (near modern Luoyang) during the Zhou dynasty. This site was excavated by the Shanxi Provincial Institute of Archaeology from 2004 to 2006. After appropriate recording, we selected 89 well-preserved human remains from 56 graves (some graves yielded several human sacrifices). A total of 197 human teeth were packed in cardboard boxes and sent to the ancient DNA laboratory of Jilin University, Changchun City, China, where they were stored in a dry and cool environment. Other skeletal samples were sent to the Research Center for Chinese Frontier Archaeology of Jilin University for anthropological analyses. The specimen numbers and the archaeological and anthropological data from the ancient individuals are shown in S1 Table.

### Ethics statement

This study was approved by the Shanxi Provincial Institute of Archaeology, NO. 33 Wenmiao Lane, Taiyuan City, Shanxi Province, China, and the State Administration of Cultural Heritage of China, NO. 10 Chaoyangmen North Street, Beijing City, China. The authors were not involved in the sample collection, and all of the samples were excavated and provided to the authors by the Shanxi Provincial Institute of Archaeology from 2004 to 2006. All of the samples were de-identified, and the specimen numbers are listed in S1 Table. All necessary permits were obtained for the described study, which complied with all relevant regulations.

### Contamination precautions and authentication criteria

All ancient DNA samples were handled with care at each step, and measures were taken to avoid contamination and ensure authenticity based on the following criteria: (1) Pre- and post-PCR analyses were conducted in two isolated laboratories, located in two separated buildings nearly one kilometer apart. The pre-PCR laboratory is one of the only molecular biology laboratories in the building. The laboratory is under positive air pressure, and the rooms for sample cleaning, DNA extraction, and PCR preparation are physically separated. Full-body protective clothing, facemasks, and gloves were used, and routine sterilization with different treatments (DNAse away, bleach, and ultraviolet light irradiation) was adopted. Researchers, after using the post-PCR laboratory, were required to avoid the pre-PCR laboratory for at least one day. (2) The tooth samples were collected as quickly and carefully as possible without washing during the excavation process and were then sent to our ancient DNA labs. The teeth were stored at -20°C. Before powdering, the teeth were soaked in 10% sodium hypochlorite solution for 2–5 min. Next, they were soaked in a 5% sodium hypochlorite solution for 20 min, rinsed with 100% ethanol, and UV-irradiated for 30 min on each side. (3) Multiple replications of mtDNA HVR-I amplification and direct sequencing, as well as Y chromosome SNP detection, were performed for each of the human remains selected for use in this study. At least two teeth were collected from each individual, and each was extracted independently. Two amplifications of each extraction were carried out on different teeth for each individual. In the extraction and amplification process, blank controls were applied for detecting contamination (one extraction blank for every five ancient samples and one PCR blank for every four reactions). (4) Teeth from ten individuals were sent to a separate laboratory (Ministry of Education Key Laboratory of Contemporary Anthropology and Center for Evolutionary Biology, Fudan University) to perform independent replications of the DNA extraction, amplification and direct sequencing (S1 Table). (5) The mtDNA HVR-I segments of these ten samples were cloned using the Promega Cloning kit (Promega, USA) to detect possible DNA damage, contamination, and jumping PCR as well as any sequencing errors (S1 Table).

### DNA extraction, amplification, cloning and sequencing

After the routine sterilization treatments described above, the tooth samples were pulverized in a cryogenic grinder (6750 Freezer Mill, SPEX, Metuchen, NJ, USA). DNA was extracted from the tooth powder using a QIAamp DNA Mini Kit (Qiagen, Germany) following the manufacturer’s protocol. Next, DNA amplification, cloning, and sequencing were conducted for subsequent mtDNA analyses. The protocols used were previously published [21].

### mtDNA analysis

The mtDNA fragment HVR-I (nucleotide positions 16035–16409) was amplified using two overlapping primer pairs and then sequenced. Haplotypes were identified by comparing the variable nucleotide positions to the revised Cambridge reference sequence (CRS) [22]. Mitochondrial haplogroups were determined using the HVR-I fragment and several SNPs on the mtDNA HVR-II fragment and in the mtDNA coding region, with classifications determined according to the East Asian mtDNA classification tree [23,24]. Haplogroups M, C, D, D4, F, G, M7, M9, and N9a were examined directly using the amplified product-length polymorphisms (APLP) method [25,26] by detecting mutations at positions 10400, 14318, 5178, 3010, 3970, 4833, 6455, 3394, and 5417, respectively. Haplogroups A, D5, M8, M10, R, and Z were identified by sequencing to detect mutations at positions 663, 10397, 14470, 10646, 12705, and 152, respectively. Haplogroup B was identified using electrophoresis to detect the presence of a CoII/tRNAlys 9-bp deletion. All primers are listed in S2 Table.

### Data analysis

A principal component analysis (PCA), shared mtDNA haplotype analysis, F_ST_ comparison and geographic mapping of maternal genetic distances were used in the mtDNA analysis. Only PCA was used in the Y chromosome analysis. For the statistical analyses, the genetic data for current populations from mainland China were retrieved from published reports (S3 and S4 Tables). Han Chinese populations were divided into NH [11,23,27–30] and SH groups [11,23,24,27,30], and separated by the previously published prominent genetic boundary [12]. Other present-day populations were also integrated into the two population groups. These populations include the northern Minorities (NM) and the southern Minorities (SM), divided according to their language classification and geographic distribution. In short, Altaic populations [29,31–36] are attributed to belong to the NM, whereas the Tibeto-Burman [27,29–33,37–40], Hmong-Mien [29,30,32,33,38,41], Daic [29,31,33,39,40,42,43], and Austro-Asiatic populations [39,40,42,43] were classed as belonging to the SM. History document showed that many northern ethnic populations had several conflicts with the Han Chinese on the northern boundary of the Central Plain region, and a part of them merged gradually into the Han Chinese from ancient to modern times[3]. In order to analyze the contribution of ancient northern ethnic populations to present-day northern Han Chinese, we retrieved the mtDNA datasets of two ancient populations-Xiongnu (XN) and Xianbei (XB). The XN data were retrieved from a 2,000-year-old necropolis in the Egyin Gol Valley of Mongolia, which yielded 46 authentic mtDNA data profiles [44]. The XB data included two ancient populations, with one excavated from a 1,600-year-old Lamadong site in the north of China [45], and the other excavated from a 1,500-year-old Qilang mountain site in the north of China [46] (Fig 1). The mtDNA haplogroups belonging to the East Eurasian pool of mtDNA lineages were integrated into the dataset for haplogroup distribution and PCA, but they were included according to their ancestral markers—A, B, C, D, F, G, M7, M8, M9, M*, N*, N9a, R, Y, and Z—as the genotyping data available in the literature are too incomplete to enable the use of more specific descendant sub-lineages. In addition, some European-specific haplogroups found at lower frequencies in current Chinese populations were pooled into the ‘West’ group, including haplogroups U, H, and J. PCA was conducted using mtDNA and Y chromosome haplogroup frequencies with SPSS 16.0 software (SPSS, Chicago, USA). The maternal genetic distances between the HB and population retrieved in this study were calculated. The maternal genetic distances between different populations were investigated using F_ST_ comparisons with Arlequin 3.11 software (http://cmpg.unibe.ch/software/arlequin3/), and the F_ST_ values were estimated using the Kimura two-parameter model [47]. This calculation was performed twice. First, the genetic distances between seven populations, including the HB, XN, XB, NH, SH, NM and SM, were calculated. Second, the genetic distances between the HB and each present-day population (including 19 Han Chinese and 40 Chinese minorities) were then calculated. Based on the F_ST_ values obtained by the second calculation, the genetic distances were mapped onto geographic maps using the Kriging algorithm from the Surfer 8.0 software (Golden Software, Colorado, USA). Arlequin sometimes yields negative F_ST_ values which are represented as equal to zero[48,49], and we did not remove the negative F_ST_ values in analyses.

## Results

### Sequence authentication

The potential for exogenous DNA contamination was minimized by the use of strict precautionary procedures in all stages of sample preparation and analysis. A total of 69 samples were amplified successfully because of the cold and dry environmental conditions, freshly excavated samples and improved methods for ancient DNA extraction [50]. Independent replicates were made for each sample, and four samples yielded amplified mtDNA HVR-I sequences that could not be reproduced, although the controls used during DNA extraction and PCR were always negative. These four samples were not used in subsequent analyses, resulting in 65 samples that yielded reproducible results. Among these samples, sample M2055ii was also discarded because it had the same mtDNA HVR-I region as that of a laboratory researcher, although consistent results were obtained from the specimen in multiple independent extractions. Most mtDNA HVR-I sequences obtained in the cloning analyses were consistent with those found by direct sequencing of PCR products (S1 Fig). The specimens derived by the cloning analyses showed appropriate ancient DNA molecular behavior. A good correlation of inferred haplogroups was found between the coding and control regions (Table 1). Thus, we consider the 64 mtDNA HVR-I sequences to be authentic.

**Table 1:** Nucleotide differences in mtDNA and Y-SNP data from Hengbei samples.

Sample^a^	Mitochondrial DNA			Y chromosome^c^	
	Haplogroup	Mutations in HVR-I (16000+)^b^	HVR-II and coding region SNPs	Haplogroup	SNPs
M1004	B	182C, 183C, 189, 217	10400C, CoII/tRNAlys 9bp deletion	-	-
M1006	F	192–304	10400C, 12705C, 3970T	O3a	M89T, M9G, M214C, M175 (5bp deletion), M122C, M324C
M1007	F	111, 129, 266, 304	10400C, 12705C, 3970T	O3a3	M89T, M9G, M214C, M175 (5bp deletion), M122C, M324C, P201C
M1009i	G	223, 362	10400T, 4833G	O3a	M89T, M9G, M214C, M175 (5bp deletion), M122C, M324C
M1009iii	D5	164, 172, 174, 182C, 183C, 189, 223, 266, 362	10400T, 5178A, 10397G	-	-
M1010	D4	209, 223, 290, 362	10400T, 5178A, 3010A	-	-
M1011v	B	111, 140, 182C, 183C, 189, 234, 243	10400C, CoII/tRNAlys 9bp deletion	-	-
M2002	D4	184, 192, 223	10400T, 5178A, 3010A	-	-
M2002i	D4	223, 362	10400T, 5178A, 3010A	-	-
M2003	A	222, 223, 290, 294, 319, 362	10400T, 663G	-	-
M2006	D4	129, 223, 256, 362	10400T, 5178A, 3010A	N	M89T, M9G, M214C, M231A
M2006i	B	93, 167, 182C, 183C, 189, 217	10400C, CoII/tRNAlys 9bp deletion	O3a	M89T, M9G, M214C, M175 (5bp deletion), M122C, M324C
M2007ii	F	129, 162, 172, 304	10400C, 12705C, 3970T	O2a	M89T, M9G, M214C, M175 (5bp deletion), M95T
M2007iii	A	93, 223, 290, 319	10400T, 663G	O2a	M89T, M9G, M214C, M175 (5bp deletion), M95T
M2021	M10	223, 311	10400T, 10646A	-	-
M2036	M	162	10400T	Q1a1	M89T, M9G, M45A, M242T, M120C
M2036i	B	136, 183C, 189, 217, 222	10400C, CoII/tRNAlys 9bp deletion	O3a	M89T, M9G, M214C, M175 (5bp deletion), M122C, M324C
M2036ii	B	93, 145, 183C, 189, 217	10400C, CoII/tRNAlys 9bp deletion	-	-
M2036iii	B	140, 172, 183C, 189, 243	10400C, CoII/tRNAlys 9bp deletion	-	-
M2044	M9a	223, 234, 248, 265T, 316, 362	10400T, 3394C	O3a3	M89T, M9G, M214C, M175 (5bp deletion), M122C, M324C, P201C
M2045	M9a	66, 223, 248, 265T, 316, 362	10400T, 3394C	Q1a1	M89T, M9G, M45A, M242T, M120C
M2049	D4	223, 362	10400T, 5178A, 3010A	Q1a1	M89T, M9G, M45A, M242T, M120C
M2049i	Z	223, 260, 298, 302	10400T, 152C	-	-
M2053	M	93, 129, 223	10400T	-	-
M2055	A	223, 290, 319, 362	10400T, 663G	O3a3	M89T, M9G, M214C, M175 (5bp deletion), M122C, M324C, P201C
M2055i	M	184, 189, 223, 316, 362	10400T	O3a3	M89T, M9G, M214C, M175 (5bp deletion), M122C, M324C, P201C
M2083	M9a	223, 265T, 316, 362	10400T, 3394C	O*	M89T, M9G, M214C, M175 (5bp deletion)
M2084	D4	93, 214, 223, 362	10400T, 5178A, 3010A	Q1a1	M89T, M9G, M45A, M242T, M120C
M2089	A	93, 223, 290, 319, 362	10400T, 663G	O*	M89T, M9G, M214C, M214C, M175 (5bp deletion)
M2107	D4	172, 174, 223, 362	10400T, 5178A, 3010A	-	-
M2129i	D5	92, 182C, 183C, 189, 223, 360, 362	10400T, 5178A, 10397G	-	-
M2129ii	C	223, 298, 327	10400T, 14318C	-	-
M2144	D4	223, 325, 362	10400T, 5178A, 3010A	-	-
M2144i	Z	185, 223, 260, 298	10400T, 152C	-	-
M2144ii	M10	66, 223, 311	10400T, 10646A	-	-
M2146i	A	223, 290, 319, 362	10400T, 663G	-	-
M2146ii	N9a	172, 223, 257A, 261	10400C, 5417A	-	-
M2146iii	D4	164, 223, 266, 362	10400T, 5178A, 3010A	-	-
M2147	D5	164, 182C, 183C, 189, 223, 266, 362	10400T, 5178A, 10397G	-	-
M2149	M9a	223, 265T, 316, 362	10400T, 3394C	-	-
M2150i	D4	223	10400T, 5178A, 3010A	-	-
M2150ii	M	174, 192, 223, 311, 320	10400T	-	-
M2155	C	298, 327	10400T, 14318C	-	-
M2158	M	184, 187, 188, 223	10400T	Q1a1	M89T, M9G, M45A, M242T, M120C
M2158ii	M10	223, 311	10400T, 10646A	-	-
M2158iii	M8	184, 223, 293, 298, 319	10400T, 14470C	O*	M89T, M9G, M214C, M175 (5bp deletion)
M2170	M8	184, 223, 293C, 298, 319	10400T, 14470C	-	-
M2286	A	126, 192A, 223, 234, 235, 290, 319	10400T, 663G	Q1a1	M89T, M9G, M45A, M242T, M120C
M3012	R	129, 223, 264, 266, 267, 304	10400C, 12705C	-	-
M3016	M	164, 172, 182C, 183C, 189, 259, 362	10400T	Q1a1	M89T, M9G, M45A, M242T, M120C
M3039	F	92, 207, 304	10400C, 12705C, 3970T	-	-
M3046	D4	223, 362	10400T, 5178A, 3010A	Q1a1	M89T, M9G, M45A, M242T, M120C
M3051	M7	71, 223, 295	10400T, 6455T	-	-
M3099	F	129, 162, 172, 304	10400C, 12705C, 3970T	-	-
M3124	F	108, 129, 162, 172, 304	10400C, 12705C, 3970T	-	-
M3147	F	261	10400C, 12705C, 3970T	-	-
M3170	B	182C, 183C, 189, 217, 261, 311	10400C, CoII/tRNAlys 9bp deletion	Q1a1	M89T, M9G, M45A, M242T, M120C
M3218	D4	223, 362	10400T, 5178A, 3010A	-	-
M3267	B	183C, 185, 189, 217	10400C, CoII/tRNAlys 9bp deletion	-	-
M3327	N9a	223, 257A, 261, 294	10400C, 5417A	Q1a1	M89T, M9G, M45A, M242T, M120C
M3336	M	93, 129, 223, 256	10400T	Q1a1	M89T, M9G, M45A, M242T, M120C
M3349	M	379	10400T	O*	M89T, M9G, M214C, M175 (5bp deletion)
M3374	M9a	223, 291, 316, 362	10400T, 3394C	O3a3	M89T, M9G, M214C, M175 (5bp deletion), M122C, M324C, P201C
M3379	A	223, 290, 362	10400T, 663G	-	-
R1	F	183C, 189, 304	10400C, 12705C, 3970T	O3a3	M89T, M9G, M214C, M175 (5bp deletion), M122C, M324C, P201C
R2	F	124, 183C, 189, 278, 293, 362	10400C, 12705C, 3970T	-	-
R3	D4	126, 174, 223, 311, 362	10400T, 5178A, 3010A	-	-
R4	F	136, 183C, 189, 217, 218, 239, 248	10400C, 12705C, 3970T	-	-
R5	D4	93, 223, 255, 362	10400T, 5178A, 3010A	-	-
R6	C	223, 298, 311, 327	10400T, 14318C	-	-
R7	B	189, 261, 278, 311, 362	10400C, CoII/tRNAlys 9bp deletion	O3a3	M89T, M9G, M214C, M175 (5bp deletion), M122C, M324C, P201C

^a^R1-R7, research laboratory staff.

^b^ Mutation sites are numbered according to the CRS. The numbers without suffixes indicate transitions, and the suffixes A, C and T indicate transversions.

^c^—indicates no data.

### Mitochondrial DNA of Hengbei ancient people

Reproducible mtDNA HVR-I sequences were obtained for 64 of the 89 individuals excavated from the Hengbei site. Very few of the analyzed samples share the same haplotype. The exceptions were five samples (M1009i, M2002i, M2049, M3046 and M3218) that yielded mutations at positions 16223 and 16362. Further analysis showed that M1009i contained an A→G transition at position 4833, and the remaining four samples contained two other mutations: a C→A transversion at position 5178 and a G→A transition at position 3010. Thus, these samples were attributed to two different haplotypes: G and D4. Finally, all samples were classified into 57 different haplotypes. Like the most of present-day populations, they yielded a high haplotype diversity (H) of 0.993 ± 0.005 (the range of haplotype diversity for the modern comparative populations: 0.857–1.000, S3 Table). When the mtDNA coding region or HVR-II SNPs were combined with the HVR-I sequence, all haplotypes were further attributed to 15 different haplogroups or sub-haplogroups—A, B, C, D4, D5, F, G, M, M7, M8, M9, M10, N9a, R and Z (Table 1)—all of which belong to the East Eurasian pool of mtDNA lineages [23,24,51].

### Distribution of mitochondrial DNA haplogroups

According to a previous study, the haplogroups of the Han Chinese can be classified into the northern East Asian-dominating haplogroups, including A, C, D, G, M8, M9, and Z, and the southern East Asian-dominating haplogroups, including B, F, M7, N*, and R [11]. These haplogroups account for 52.7% and 33.85% of those in the NH, respectively. Among these haplogroups, D, B, F, and A were predominant in the NH, with frequencies of 25.77%, 11.54%, 11.54%, and 8.08%, respectively [11,23,24,28,51]. However, in the SH, the northern and southern East Asian-dominating haplogroups accounted for 35.62% and 51.91%, respectively. The frequencies of haplogroups D, B, F, and A reached 15.68%, 20.85%, 16.29%, and 5.63%, respectively. Notably, in the HB samples, haplogroups D, B, F, and A were also predominant and showed frequencies of 23.44%, 12.5%, 10.93%, and 10.93%, respectively. In addition, the frequency of haplogroup M* was high and reached 17.19%. Other haplogroups such as C, G, M7, M8, M9, Z, N9a and R had lower frequencies at 3.13%, 1.56%, 1.56%, 3.13%, 7.81%, 3.13%, 3.13% and 1.56%, respectively. The northern and southern East Asian-dominating haplogroups account for 50.04% and 26.56%, respectively, which is similar to the values in the NH (S2 Fig).

### Principal component analysis

**Fig 2:**
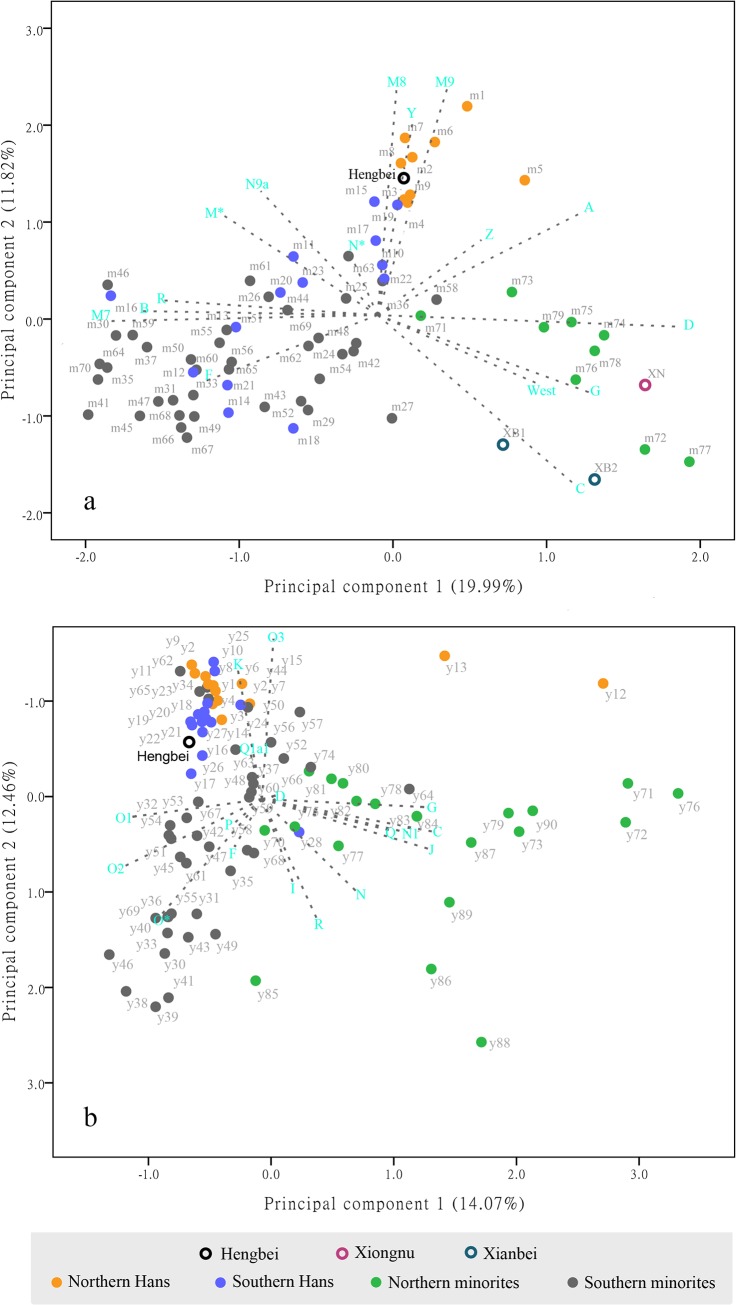
Principal component plot of ancient and present-day Chinese populations. (a) mtDNA haplogroup frequency plot. For each population’s code, see S3 Table. (b) Y-chromosome haplogroup frequency plot. For each population’s code, see S4 Table.

To further identify the genetic affinities among the HB, two ancient populations and the present-day Chinese population, represented by 9 NH, 9 NM, 14 SH and 57 SM groups, the mtDNA haplogroup distributions were compared using a PCA. The PCA plot of the first and second components (31.81% of the total variance, Fig 2A) shows that the current populations largely segregate into three main clusters: NH (in orange), SH (in blue) and SM (in gray), and NM (in green). The distribution of populations in the PCA plot was in line with their geographic distribution, and these populations were separated by the first principal component. The populations living in northern China (NH and NM) are located on the right side of the PCA, and they contain the northern East Asian-dominating haplogroups A, C, D, G, M8, M9, and Z. In contrast, the populations living in southern China (SH and SM) are located on the left side of the PCA, and they contain the southern East Asian-dominating haplogroups B, F, M7, and R. Moreover, the NH can be separated from other populations except for two SH (Hubei and Shanghai), using the second principal component. The HB population (PC1 value: 0.071; PC2 value: 1.453) groups closely with the NH (PC1 value: 0.239±0.269; PC2 value: 1.590±0.336). Overall, these results indicate that the HB population shares a similar genetic profile with the NH that is distinct from the NM and ancient northern ethnic groups.

In the Y chromosome SNP analysis, the PCA plot of components 1 and 2 (26.53% of the total variance, Fig 2B) shows that the current populations largely segregate into three main clusters: NH and SH (in orange and blue), SM (in gray), and NM (in green). Northern minorities can be separated from the other populations by the first principal component, and they contain the northern East Asian-dominating haplogroups N and C. NH and SH are clustered together and contain haplogroups O3 and Q1a1, and they are separated from other populations by the second principal component. The HB (PC1 value:-0.373; PC2 value:-0.678) are grouped closely with the NH and SH cluster (PC1 value: 0.054±0.706; PC2 value: -0.911±0.371), which suggests that the HB and present-day Han Chinese shared close paternal lineages.

### Haplotype-sharing analysis

**Fig 3:**
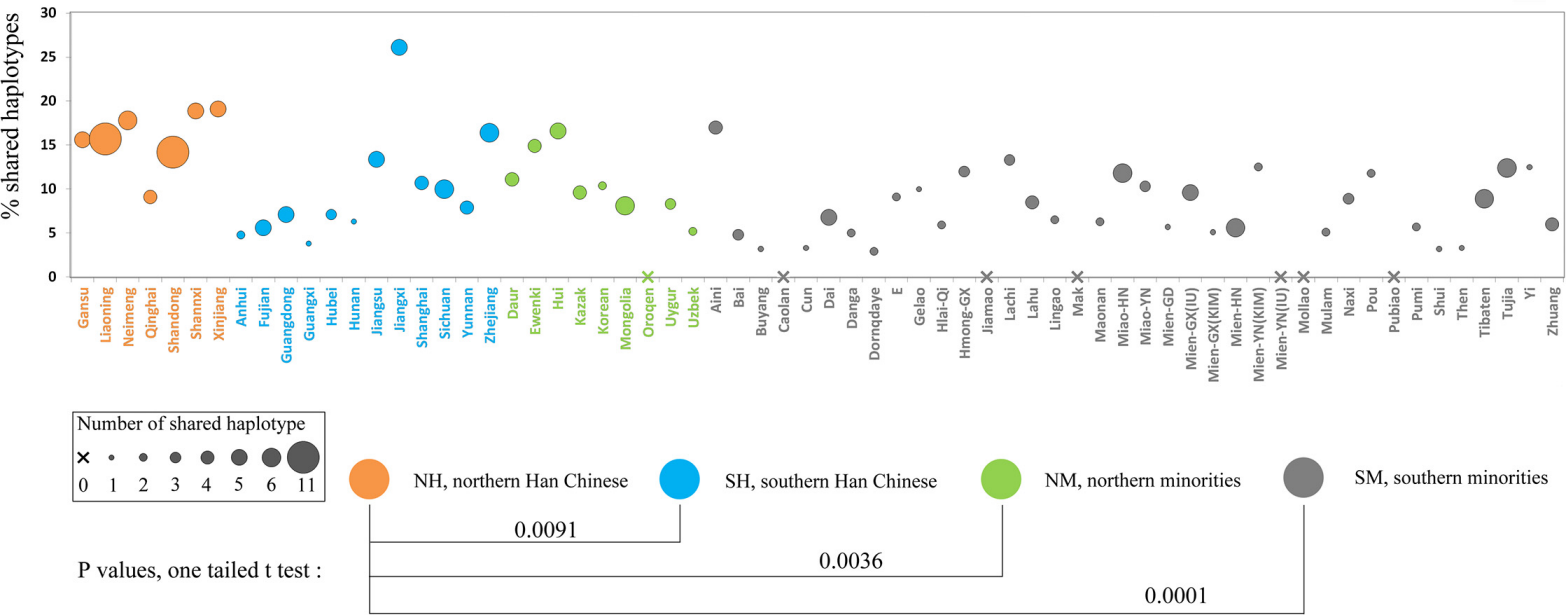
Percentage of haplotypes found in Hengbei individuals and matched in various present-day populations of China.

The datasets from current populations of China and the two ancient populations were retrieved to search for identical matches with each HB haplotype. A subset of 25 of 57 haplotypes from the HB population matched the retrieved populations. Notably, NH and HB shared the most haplotypes. The percentage of shared haplotypes in the NH pool was significantly higher than in other current population pools (P values of the t-test for the NH/SH, NH/NM and NH/SM were 0.0091, 0.0036 and 0.0001, respectively) (Fig 3).

### Analysis of the spatial distribution of the genetic matrilineal distances

**Fig 4:**
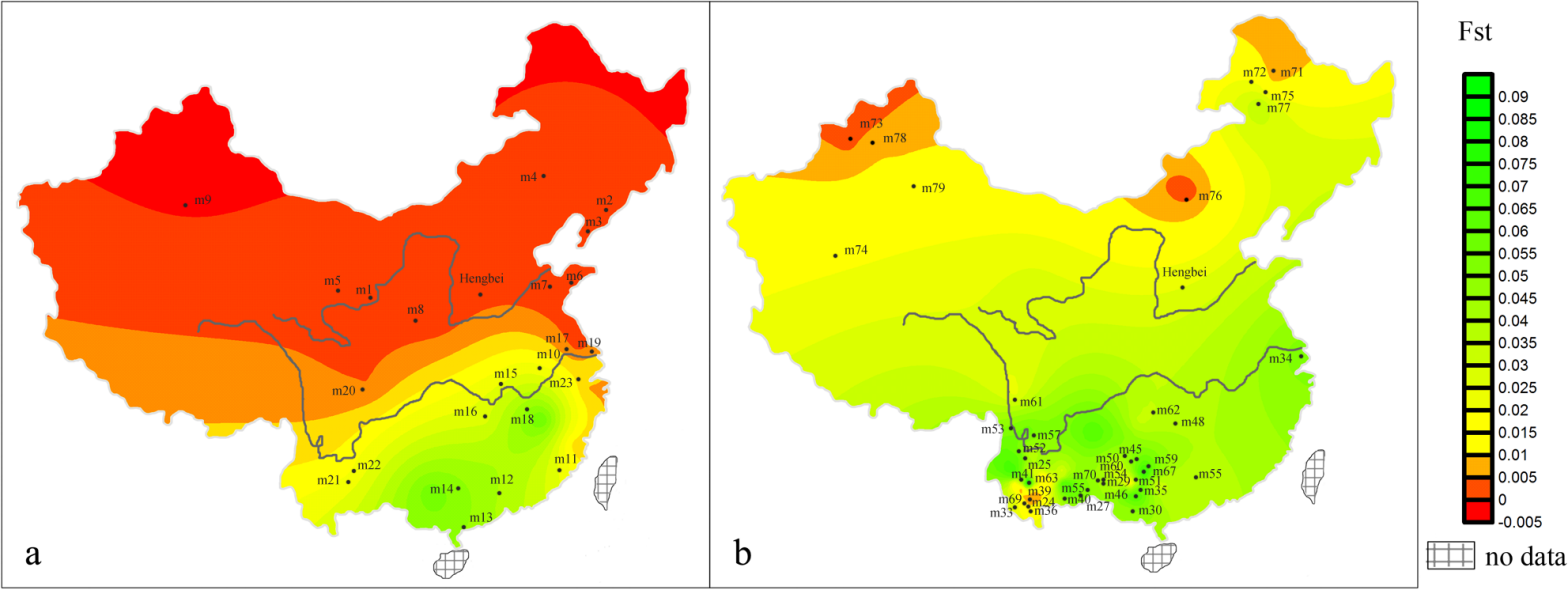
The genetic matrilineal distances between HB and current Chinese populations. (a) F_ST_ values between HB and the Han Chinese. (b) F_ST_ values between HB and the minorities of China. For each population’s code, see S3 Table.

F_ST_ comparisons were used to evaluate the maternal genetic differentiation between the HB and other relative populations. The F_ST_ between HB and NH was the lowest (-0.00183) and had a p value larger than 0.05, whereas the F_ST_ values of HB/SH (0.00998), HB/NM (0.00321) and HB/SM (0.02355) were much larger than that of HB/NH (S3 Fig), indicating that the HB population was extremely similar to the NH population. To further analyze the relationship between the ancient people and the Han Chinese, an analysis of the spatial distribution of the genetic matrilineal distances using F_ST_ statistics as a genetic distance measure was conducted. The results show that there is a significant boundary in the spatial maps based on F_ST_ values that is consistent with the genetic boundaries between NH and SH (Fig 4A) [12]. Almost all of the F_ST_ values between the HB and NH (range: -0.00222 to 0.01922) were negative and had p values greater than 0.05, whereas most of the F_ST_ values between the HB and SH were high (range: -0.00024 to 0.05291) and had p values less than 0.05 (S5 Table). A one-tailed t-test showed that the F_ST_ values for HB/NH were significantly lower than that of HB/SH (p value of 0.0032). Moreover, the genetic distance maps for HB and the minorities exhibits a green shade (Fig 4B), and most of the p values between these two groups are less than 0.01 (S5 Table). This pattern indicates that HB is closer to NH than to other Chinese populations.

## Discussion

The Han Chinese originated from the Central Plain region, which is substantially smaller than the region the Han Chinese now occupy. According to historical documents, the Han Chinese suffered many conflicts with natives prior to expansion into their lands[3]. The Han migrated northward into regions inhabited by many ancient northern ethnic groups. Based on the advanced agriculture, technology, and culture, the Han Chinese or their ancestors often had a greater demographic advantage over ancient northern ethnic groups. Thus, the Han Chinese or their ancestors might have played a predominant role in the genetic mixture of populations. This scenario would mean that the genetic structure of the NH was shaped a long time ago. In our study, the HB population showed great genetic affinities with the NH when maternal lineages were tested. First, the HB contained a distribution and component of mtDNA similar to that of the NH and clustered closely together with the NH in the PCA plot. Second, the HB shared more haplotypes with the NH than with other populations in the haplotype-sharing analysis. Third, the F_ST_ value from comparisons between the HB and NH populations was lowest and negative. Generally, F_ST_ value should theoretically range between 0 and 1. However, if the estimate of within diversity is larger than the estimate obtained of variance among groups, negative F_ST_ values should be obtained, and they are represented as equal to zero[48,49]. It indicated that HB bore a very high similarity to NH populations. Considering the location and culture of the HB, we suggest that the NH might have provided a significant contribution to the HB and find that the maternal genetic profiles of the NH were shaped 3,000 years ago.

These conclusions are further supported by the relationship between the HB and NM, XN, and XB. In our study, the PCA plot is consistent with the SH not only mixing with the SM but also with the NH, which is consistent with a previous genetic study that concluded that the SH was formed from almost equal contributions of southward migrating Han Chinese and southern natives [11]. However, the NH and NM group into two separate clusters, which is not consistent with their current geographic distributions because these two populations often live together in the northern region of China. Moreover, XN,XB1 and XB2 pool into the NM and are far away from HB and NH. A haplotype-sharing analysis of the three ancient populations and each present-day Han Chinese population shows that the fraction of haplotypes from HB is significantly higher than that from XN, XB1 and XB2 (all of the p values of HB/XN, HB/XB1 and XB2 are less than 0.01, two-tailed t-test; S4 Fig). In the F_ST_ comparisons, the F_ST_ values of the XN/HB, XB/HB, XB/NH, XN/NH, and NM/NH are significantly higher, and all of the p values are less than 0.05, indicating that the XN and XB were distinct from the NH and HB (S3 Fig). This finding indicates that the ancient populations of the XN and XB had a limited maternal genetic impact on present-day Han Chinese.

Y chromosome SNP analysis was consistent with the conclusions drawn from studying the maternal lineages. In the paternal lineage, HB contained the haplogroups or sub-haplogroups N, O*, O2a, O3 and Q1a1. The total frequencies of these haplogroups reached high levels (66%–100%) in current Han Chinese [11,27,30,52,53]. Haplogroup Q1a1, which was predominant in HB, is highly specific to the Han Chinese [53]. Haplogroup O3, the second highest frequency (33.34%) in HB, occupies the highest frequencies in almost all current Han Chinese populations (32.5%-76.92%) [11,27,30,52,53]. Moreover, in the PCA plot, HB groups closely with the Han Chinese. These results indicate that the 3,000-year-old ancient people from the Central Plain region share similar paternal genetic profiles with the current Han Chinese. In contrast, XN yielded three haplogroups (N3, Q, and C) but no haplogroup O [54]. The frequency of O in NM is significantly lower than the frequency of O in NH, but the frequency of haplogroup N shows the inverse trend. Moreover, NM has a relatively high frequency of haplogroup R, but NH does not. Thus, the major paternal genetic component of NH was shaped in the Central Plain region of China prior to 3,000 years ago.

According to historical documents, most of the ancient populations that inhabited the northern region of China were nomads. With no permanent settlement, these populations often moved from place to place. In contrast, the ancestors of the Han Chinese were farming people, who often settled down in a region and seldom moved. Following increases in population size, the ancestors of the Han Chinese gradually expanded into the surrounding areas and conflicted with the ancient northern groups. Finally, most of the ancient northern groups gradually disappeared. Because of the large differences in lifestyle and culture between farmers and nomads, most of the ancient northern ethnic populations might have migrated to other areas when they were defeated, and their lands were gradually occupied by the Han Chinese. A similar population replacement model is also found in Europe, where the diffusion of agriculture and language from the Near East was concomitant with a large movement of farmers [13,55–58]. The Han Chinese have the largest population size relative to the populations they admixed with, suggesting a stable genetic structure in the northern Han Chinese for at least the past 3,000 years.

## Supporting Information

S1 Fig Alignment of cloned mtDNA sequences from ten samples. The primer sequences are shadowed.
(TIF)

S2 Fig mtDNA haplogroups of ancient and present-day Chinese populations. (TIF)

S3 Fig Population pairwise F_ST_s (below the diagonal) and matrix of significant F_ST_ p values (above the diagonal). HB, ancient Hengbei people; XN, Xiongnu; XB, Xianbei; NH, northern Han; SH, southern Han; NM, northern Minorities; SM, southern Minorities.
(TIF)

S4 Fig Percentages of mtDNA haplotypes shared between ancient Chinese populations and present-day Han Chinese populations. (TIF)

S1 Table Characteristics of samples excavated from the Hengbei site. (PDF)

S2 Table Primers used in this study. (PDF)

S3 Table Estimated percentages of mtDNA haplogroups shared among ancient populations and modern Chinese populations as well as the genetic diversity of each population. (PDF)

S4 Table Estimated percentages of Y chromosome haplogroups shared among HB and modern Chinese populations. (PDF)

S5 Table Population pairwise F_ST_ values and F_ST_ p values between ancient and current populations. (PDF)
