# Supplementary figures and images for: Ancient DNA Reveals That the Genetic Structure of the Northern Han Chinese Was Shaped Prior to 3,000 Years Ago
Source: PLoS One. 2015 May 4;10(5):e0125676. doi: 10.1371/journal.pone.0125676 (PMC4418768; doi:10.1371/journal.pone.0125676)

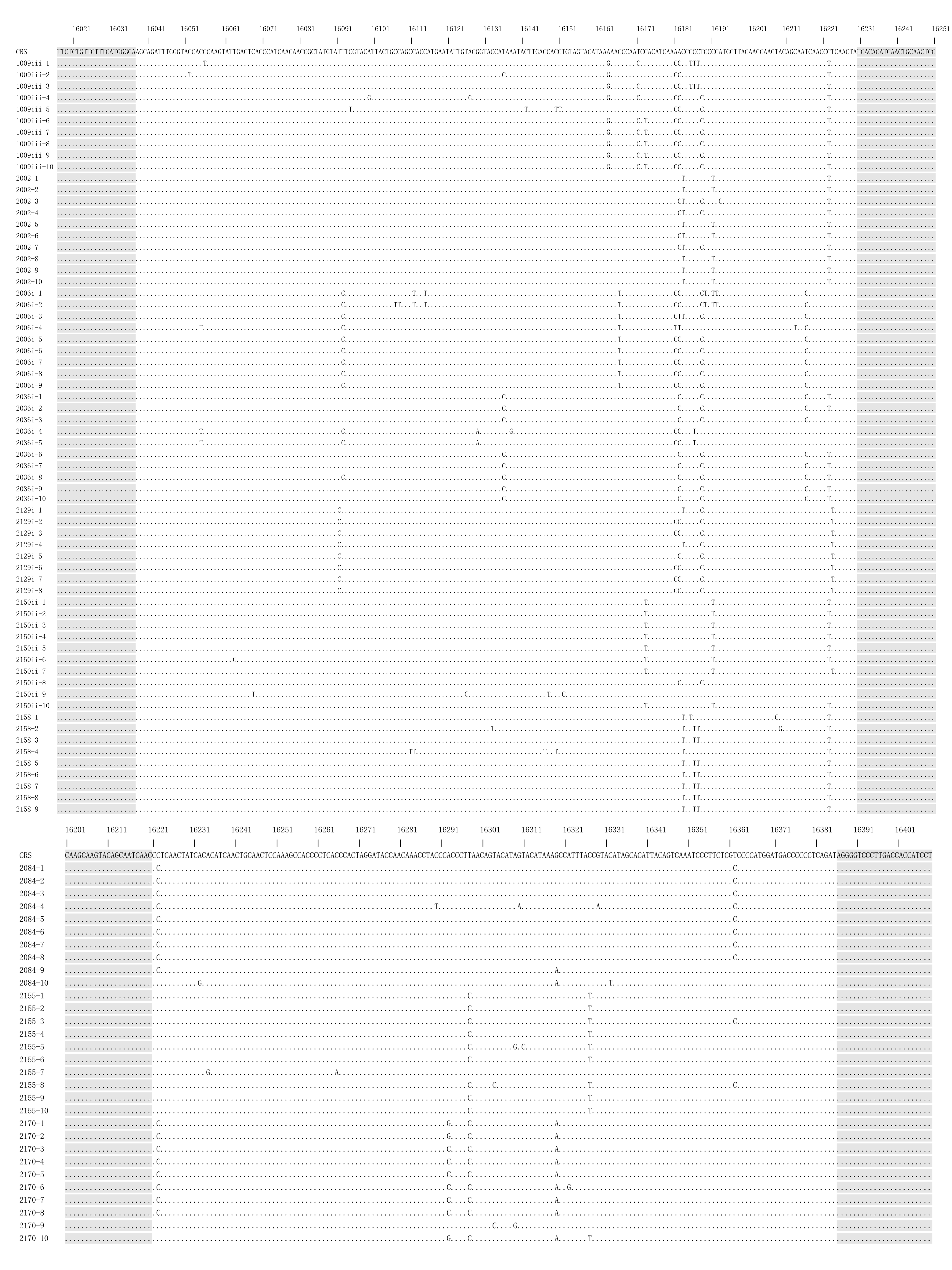

Supplement: S1 Fig — The primer sequences are shadowed. (TIF) [file pone.0125676.s001.tif]

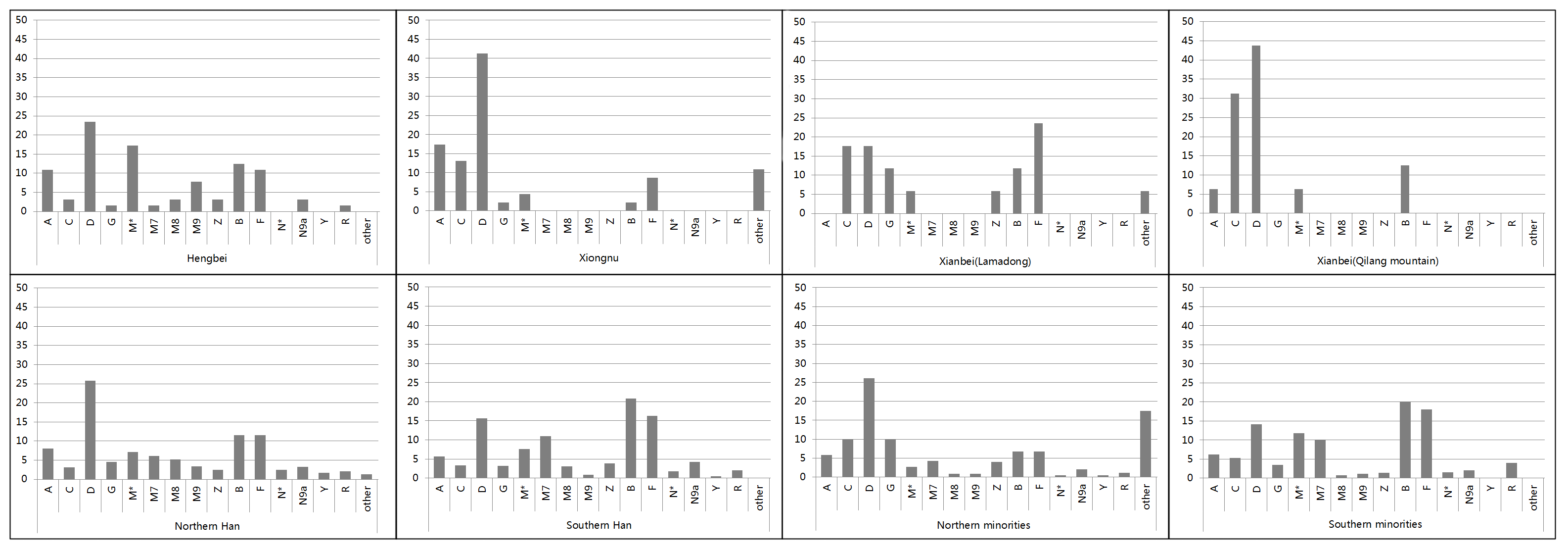

Supplement: S2 Fig — (TIF) [file pone.0125676.s002.tif]

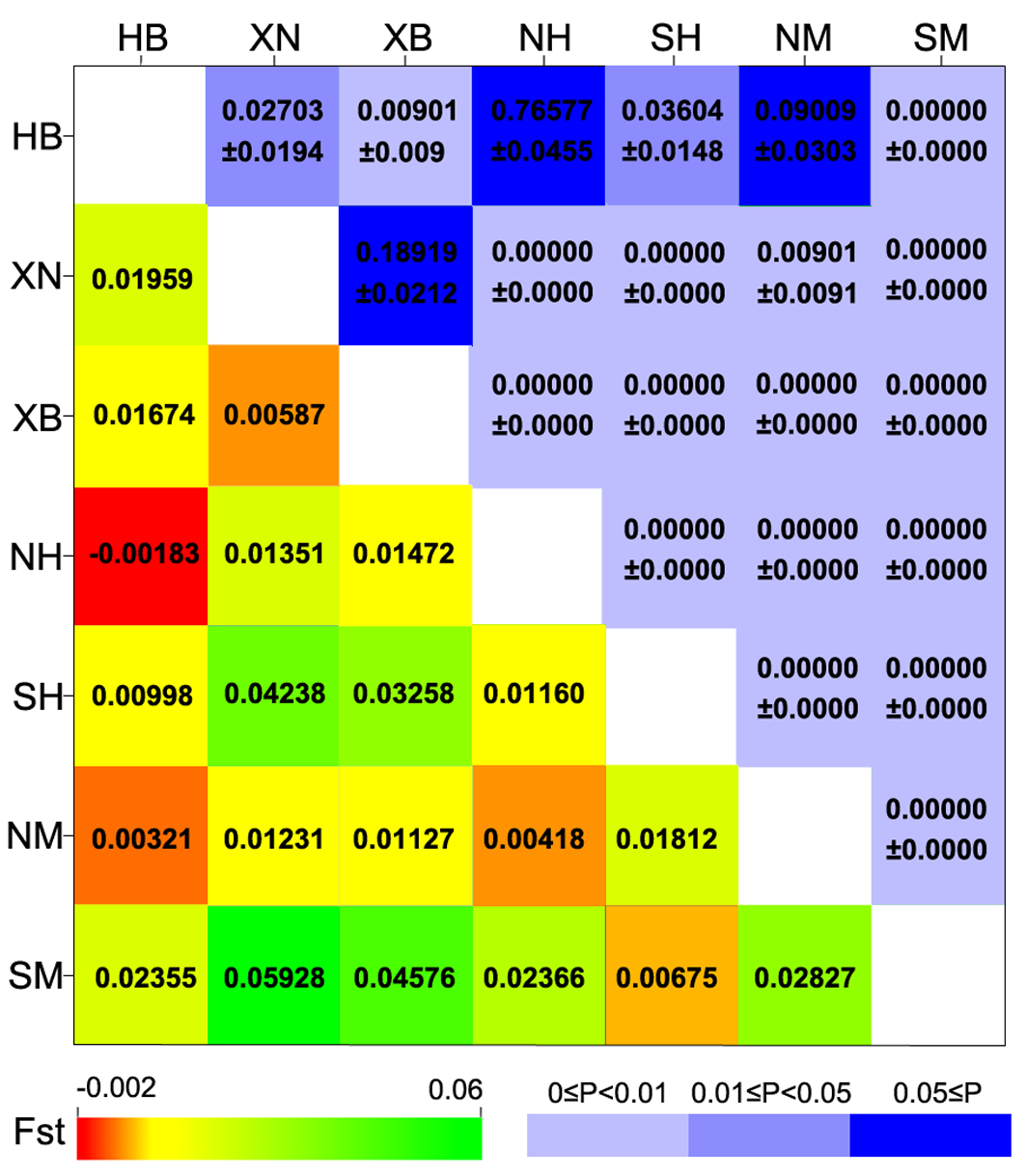

Supplement: S3 Fig — HB, ancient Hengbei people; XN, Xiongnu; XB, Xianbei; NH, northern Han; SH, southern Han; NM, northern Minorities; SM, southern Minorities. (TIF) [file pone.0125676.s003.tif]

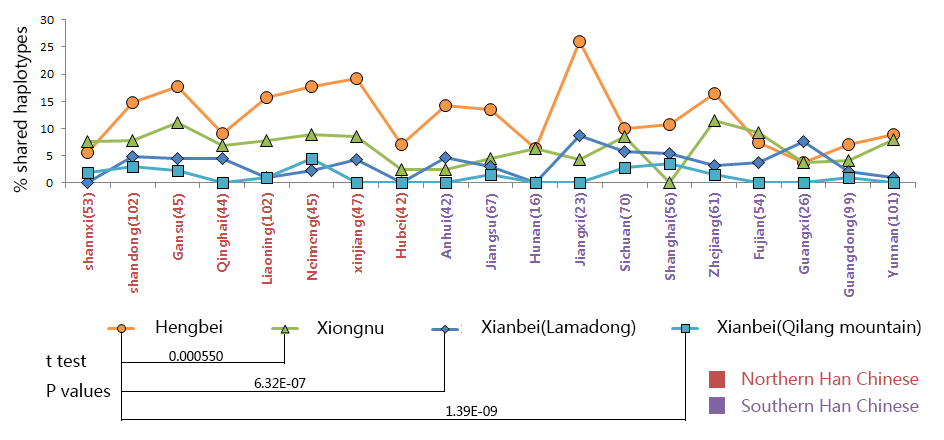

Supplement: S4 Fig — (TIF) [file pone.0125676.s004.tif]
