# Supplementary material for: Ancient DNA Reveals That the Genetic Structure of the Northern Han Chinese Was Shaped Prior to 3,000 Years Ago
Source: PLoS One. 2015 May 4;10(5):e0125676. doi: 10.1371/journal.pone.0125676 (PMC4418768; doi:10.1371/journal.pone.0125676)
Supplement: S1 Table — (PDF) [file pone.0125676.s005.pdf]

Table S1 Characteristics of samples excavated from the Hengbei site.

Burial number	Sample number	Sample information	Age at death	Sex		Mitochondrial DNA amplification	Y chromosome SNP
				Morphology	Molecular		
M1002	M1002	Tomb master	30±	Female	Fail	Fail	-
M1004	M1004	Tomb master	25-30	Male	Male	Success	Fail
M1006	M1006[a]	Tomb master	40±	Male	Male	Success	Success
	M1006i	Immolated slave	13-14	?	Fail	Fail	-
M1007	M1007	Tomb master	25±	Male	Male	Success	Success
M1009	M1009	Tomb master	?	?	Fail	Fail	-
	M1009i	Immolated slave	11-12	?	Male	Success	Success
	M1009iii[ab]	Immolated slave	16-17	Female	Female	Success	-
M1010	M1010	Tomb master	30±	Female	Female	Success	-
M1011	M1011	Tomb master	?	?	Fail	Fail	-
	M1011i	Immolated slave	25-30	Female	Fail	Fail	-
	M1011ii	Immolated slave	16-17	?	Fail	Fail	-
	M1011iii	Immolated slave	30-35	Male	Fail	Fail	-
	M1011v	Immolated slave	16-17	Male	Fail	Success	-
M2002	M2002[b]	Tomb master	34-36	Male	Female	Success	-
	M2002i	Immolated slave	16-17	Male	Female	Success	Fail
M2003	M2003	Tomb master	35-40	Female	Female	Success	-
M2006	M2006[a]	Tomb master	45±	Male	Male	Success	Success
	M2006i[b]	Immolated slave	13-14	?	Male	Success	Success
M2007	M2007	Tomb master	?	?	Fail	Fail	-
	M2007ii	Immolated slave	adult	Male	Male	Success	Success
	M2007iii	Immolated slave	25-30	Male	Male	Success	Success
M2021	M2021	Tomb master	25±	Female	Female	Success	-
	M2021i	Immolated slave	20±	Female	Fail	Fail	-
M2036	M2036	Tomb master	30-35	Male	Male	Success	Success
	M2036i[b]	Immolated slave	13-14	?	Male	Success	Success
	M2036ii	Immolated slave	17-18	Male	Female	Success	Fail
	M2036iii	Immolated slave	12-13	?	Female	Success	-
M2044	M2044	Tomb master	45±	Male	Male	Success	Success
M2045	M2045	Tomb master	30±	Male	Male	Success	Success
M2049	M2049	Tomb master	30-35	Male	Male	Success	Success
	M2049i	Immolated slave	20±	Female	Female	Success	-
M2053	M2053	Tomb master	16-17	Female	Female	Success	-
M2055	M2055	Tomb master	35-40	Male	Male	Success	Success
	M2055i	Immolated slave	25-30	Male	Male	Success	Success
	M2055ii	Immolated slave	14-15	?	Female	Showed the same sequence as the researcher	-
M2060	M2060	Tomb master	30-35	?	Fail	Fail	-
M2077	M2077	Tomb master	20-25	Male	Fail	Fail	-
M2083	M2083	Tomb master	25±	Male	Male	Success	Success
M2084	M2084[a,b]	Tomb master	30±	Male	Male	Success	Success
M2089	M2089	Tomb master	?	?	Male	Success	Success
M2107	M2107	Tomb master	16-17	Female	Female	Success	-
M2129	M2129	Tomb master	21-29	Female	Fail	Fail	-
	M2129i[b]	Immolated slave	14-15	?	Female	Success	-
	M2129ii	Immolated slave	6-7	?	Female	Success	-
	M2129iii[a]	Immolated slave	16-17	Male	Fail	No consistent result obtained by replication	-
M2144	M2144	Tomb master	25-30	Female	Female	Success	-
	M2144i	Immolated slave	11-12	?	Fail	Success	-

	M2144ii	Immolated slave	14-15	?	Fail	Success	-
M2146	M2146	Tomb master	?	?	Fail	Fail	-
	M2146i	Immolated slave	5±	?	Fail	Success	-
	M2146ii	Immolated slave	10-11	?	Fail	Success	-
	M2146iii	Immolated slave	20±	Female	Fail	Success	-
	M2146iv	Immolated slave	30-35	Female	Fail	Fail	-
M2147	M2147	Tomb master	16-17	Female	Female	Success	-
M2149	M2149	Tomb master	25	Female	Female	Success	-
M2150	M2150	Tomb master	?	?	Fail	Fail	-
	M2150i	Immolated slave	25±	Male	Fail	Success	-
	M2150ii[b]	Immolated slave	22±	Female	Female	Success	-
	M2150iii	Immolated slave	20-25	Male	Fail	Fail	-
M2155	M2155[b]	Tomb master	35-40	Male	Female	Success	Fail
M2158	M2158[b]	Tomb master	35-40	Male	Male	Success	Success
	M2158i	Immolated slave	25-30	Female	Fail	Fail	-
	M2158ii	Immolated slave	10±	?	Fail	Success	-
	M2158iii	Immolated slave	25-30	Male	Male	Success	Success
M2159	M2159	Tomb master	25-30	Male	Fail	Fail	-
M2161	M2161	Tomb master	?	?	Fail	Fail	-
M2170	M2170[b]	Tomb master	16-17	Male	Female	Success	Fail
M2286	M2286	Tomb master	?	?	Male	Success	Success
M3005	M3005[a]	Tomb master	20	Female	Female	No consistent result obtained by replication	-
M3012	M3012	Tomb master	25	Male	Male	Success	Fail
M3016	M3016	Tomb master	20-25	Male	Male	Success	Success
M3027	M3027	Tomb master	30	Male	Male	No consistent result obtained by replication	-
M3039	M3039[a]	Tomb master	20	Female	Female	Success	-
M3046	M3046[a]	Tomb master	45-50	Male	Male	Success	Success
M3051	M3051	Tomb master	25-30	Female	Female	Success	-
M3095	M3095	Tomb master	25-30	Female	Fail	Fail	-
M3099	M3099	Tomb master	30	Female	Female	Success	-
M3124	M3124	Tomb master	14-15	?	Female	Success	-
M3147	M3147	Tomb master	20-25	Male	Male	Success	Fail
M3170	M3170[a]	Tomb master	20-25	Male	Female	Success	Fail
M3199	M3199	Tomb master	20	Female	Female	No consistent result obtained by replication	-
M3218	M3218	Tomb master	30	Male	Fail	Success	-
M3267	M3267	Tomb master	30	Female	Female	Success	-
M3327	M3327	Tomb master	16-17	Male	Male	Success	Success
M3336	M3336	Tomb master	20	Male	Male	Success	Success
M3349	M3349[a]	Tomb master	45-50	Male	Male	Success	Success
M3374	M3374	Tomb master	30	Male	Male	Success	Success
M3379	M3379	Tomb master	30	Female	Female	Success	-

[a] The samples were replicated independently at Fudan University.

[b] mtDNA HVR-I segments cloned from samples.

-, not analyzed; ?, unknown.
