# Supplementary material for: Ancient DNA Reveals That the Genetic Structure of the Northern Han Chinese Was Shaped Prior to 3,000 Years Ago
Source: PLoS One. 2015 May 4;10(5):e0125676. doi: 10.1371/journal.pone.0125676 (PMC4418768; doi:10.1371/journal.pone.0125676)
Supplement: S2 Table — (PDF) [file pone.0125676.s006.pdf]

Table S2 Primers used in this study.

| mtDNA HVR-I fragments/ haplogroups | Primers | Sites | Length (bp) |
|---|---|---|---|
| HVR I-a | L16017 5'-TTCTCTGTTCTTTCATGGGGA-3'<br>H16251 5'-GGAGTTGCAGTTGATGTGTGA-3' | | 235 bp |
| HVR I-b | L16201 5'-CAAGCAAGTACAGCAATCAAC-3'<br>H16409 5'-AGGATGGTGGTCAAGGGA-3' | | 209 bp |
| 393bp | L16017 5'-TTCTCTGTTCTTTCATGGGGA-3'<br>H16409 5'-AGGATGGTGGTCAAGGGA-3' | | 393bp |
| A | L587 5'-TTACCTCCTCAAAGCAATACA-3'<br>H761 5'-CTTGATGCTTGTCCCTTTT-3' | 663G/A | 175 bp |
| B | L587 5'-TTACCTCCTCAAAGCAATACA-3'<br>H761 5'-CTTGATGCTTGTCCCTTTT-3' | CoII/tRNAlys 9-bp deletion | 121 bp/112 bp (B) |
| C | L14318T 5'-CCTTCATAAATTATTCAGCTTCCaACACTAT-3'<br>L14318C 5'-aaaaagctaCATAAATTATTCAGCTTCCTACtCTAC-3'<br>H14318R 5'-TTAGTGGGGTTAGCGATGGA-3' | 14318C/T | 110 bp/115 bp (C) |
| D | 5178A 5'-TGATCAACGCACCTGAAACAAGA-3'<br>5178C 5'-GTCGCACCTGAAGCAAGC-3'<br>5178R 5'-CCCATTTGAGCAAAAAGCC-3' | 5178A/C | 107 bp (D)/102 bp |
| D4 | 3010A 5'-gctacaTGGATCAGGACAaCCCA-3'<br>3010G 5'-aTTGGATCAGGACtTCCCG-3'<br>3010R 5'-tTCCGGTCTGAACTCAGATC-3' | 3010A/G | 95 bp (D4)/91 bp |
| D5 | L10262 5'-ATTGCCCTCCTTTTACCCCTAC-3'<br>H10475 5'-GGGGCATTTGGTAAATATGATTATC-3' | 10397G/A | 214 bp |
| F | 3970T 5'-taaaaTGTATTCGGCTATGAAGAtTAA-3'<br>3970C 5'-GTGTATTCGGCTATGAAGtATAG-3'<br>3970R 5'-AGTCTCAGGCTTCAACATCG-3' | 3970T/C | 70 bp (F)/66 bp |
| G | 4833A 5'-tcaaaCAGAGGTTACCCtAGGCA-3'<br>4833G 5'-CAGAGGTTACCCAtGGCG-3'<br>4833R 5'-CTTACGTTTAGTGAGGGAGAG-3' | 4833G/A | 114 bp/109 bp (G) |
| M | 10400T 5'-taattaTACAAAAAGGATTAGACTGtgCT-3'<br>10400C 5'-TACAAAAAGGATTAGACaGAACC-3'<br>10400R 5'-GAAGTGAGATGGTAAATGCTAG-3' | 10400T/C | 149 bp (M)/142 bp |
| M7 | 6455T 5'-acTGTGATTAGGACGGATCtGACA-3'<br>6455C 5'-GATTAGGACGGAaCAGACG-3'<br>6455F 5'-TAGGaGCCATCAATTTCATC-3' | 6455T/C | 100 bp (M7)/95 bp |
| M8 | L15390 5'-CGATAAAATCACCTTCCACCCT-3'<br>H15570 5'-TAGGAAATATCATTCGGGCTTG-3' | 15487T/A | 181 bp |
| M9 | 3394C 5'-CGAACGAAAAATTCTTGGCC-3'<br>3394T 5'-AATACGAACGAAAAATACTAGGCT-3'<br>3394R 5'-GTCAGCGAAGGGTTGTAG-3' | 3394C/T | 88 bp (M9)/84 bp (M9) |
| M10 | L10600 5'-CTACTCTCATAACCCTCAAC-3' | 10646A/G | 163 bp |

| | | | |
|---|---|---|---|
| | H10762 5’-CATTGGAGTAGGTTTAGG-3’ | | |
| N9a | L5334 5’-AACCTCTACTTCTACCTACG-3’ | 5417A/G | 164 bp |
| | H5497 5’-ATAAAAGGGGAGATAGGTAG-3’ | | |
| R | L12604 5’-ATCCCTGTAGCATTGTTCG-3’ | 12705C/T | 151 bp |
| | H12754 5’-GTTGGAATAGGTTGTTAGCG-3’ | | |
| Z | L48 5’-CATTTGGTATTTTCGTCTGGG-3’ | 152C/T | 158 bp |
| | H205 5’-CGCTTTGGTAAGTATGTTCGC-3’ | | |
