# Supplementary material for: Ancient DNA Reveals That the Genetic Structure of the Northern Han Chinese Was Shaped Prior to 3,000 Years Ago
Source: PLoS One. 2015 May 4;10(5):e0125676. doi: 10.1371/journal.pone.0125676 (PMC4418768; doi:10.1371/journal.pone.0125676)
Supplement: S3 Table — (PDF) [file pone.0125676.s007.pdf]

Table S3 Estimated percentages of mtDNA haplogroups shared among ancient populations and modern Chinese populations, as well as haplotype diversity of each population.

Group	Population	code	size	Estimated percentage of mtDNA Haplogroup (%)																Reference	haplotype diversity(SD)
				A	B	C	D	F	G	M	M7	M8	M9	N	N9a	R	Y	Z	west		
Ancient people	Hengbei	HB	64	10.93	12.5	3.13	23.44	10.93	1.56	17.19	1.56	3.13	7.81	0	3.13	1.56	0	3.13	0	Present study	0.993(0.005)
	Xiongnu	XN	46	17.39	2.17	13.04	41.3	8.7	2.19	4.35	0	0	0	0	0	0	0	0	10.87	[1]	0.974(0.010)
	Xianbei(Lamadong)	XB1	17	0.0	11.76	17.65	17.65	23.53	11.76	5.88	0.0	0.0	0.0	0.0	0.0	0.0	0.0	5.88	5.88	[2]	1.000(0.020)
	Xianbei(Qilang mountain)	XB2	16	6.25	12.5	31.25	43.75	0.0	0.0	6.25	0.0	0.0	0.0	0.0	0.0	0.0	0.0	0.0	0.0	[3]	0.975(0.029)
Northern Han	Gansu	m1	45	17.78	0	0	20	11.11	2.22	20	6.67	4.44	2.22	6.67	0	0	2.22	4.44	2.22	[4]	0.992(0.007)
	Liaoning1	m2	51	5.88	17.65	1.96	23.53	5.88	7.84	7.84	7.84	7.84	3.92	0	1.96	1.96	1.96	1.96	1.96	[4]	0.987(0.010)
	Liaoning2	m3	51	5.88	13.73	1.96	23.53	11.76	5.88	3.92	5.88	7.84	1.96	5.88	3.92	1.96	0	3.92	1.96	[5]	0.997(0.005)
	Neimeng	m4	45	4.44	8.89	8.89	22.22	13.33	2.22	6.67	11.11	2.22	4.44	2.22	6.67	0	2.22	2.22	2.22	[4]	0.995(0.006)
	Qinghai	m5	44	9.09	4.55	4.55	31.82	4.55	4.55	9.09	4.55	6.82	4.55	4.55	2.27	0	0	2.27	6.82	[4]	0.996(0.006)
	Shandong1	m6	50	4	12	0	36	12	6	4	4	8	4	0	6	2	2	0	0	[5]	0.995(0.005)
	Shandong2	m7	76	7.89	18.42	3.95	19.74	14.47	5.26	3.95	5.26	3.95	3.95	0	2.63	2.63	3.95	3.95	0	[6]	0.998(0.003)
	Shannxi	m8	53	7.55	9.43	1.89	26.42	13.21	3.77	11.32	5.66	1.89	5.66	7.55	1.89	1.89	1.89	0	0	[4]	0.999(0.004)
	Xinjiang	m9	47	10.64	8.51	6.38	25.53	14.89	2.13	2.13	10.64	4.26	4.26	0	0	6.38	2.13	2.13	0	[5]	0.994(0.007)
Southern Han	Anhui	m10	42	11.9	21.43	0	16.67	11.9	9.52	4.76	4.76	4.76	0	2.38	4.76	2.38	0	4.76	0	[4]	0.995(0.006)
	Fujian	m11	54	0	14.81	3.7	12.96	24.07	1.85	5.56	18.52	3.7	0	0	5.56	0	1.85	7.41	0	[4]	0.997(0.004)
	Guangdong1	m12	70	0	30.88	0	14.71	26.47	1.47	5.88	11.76	2.94	0	1.47	1.47	2.94	0	0	0	[7]	0.995(0.004)
	Guangdong2	m13	30	0	20	3.33	16.67	23.33	0	23.33	3.33	0	3.33	0	6.67	0	0	0	0	[5]	0.995(0.011)
	Guangxi	m14	26	3.85	23.08	11.54	7.69	11.54	7.69	3.85	15.38	0	0	3.85	3.85	7.69	0	0	0	[4]	1.000(0.011)
	Hubei	m15	42	16.67	19.05	2.38	9.52	16.67	2.38	0	9.52	7.14	0	2.38	7.14	0	0	7.14	0	[5]	1.000(0.005)
	Hunan	m16	16	6.25	18.75	0	12.5	0	0	25	12.5	0	0	0	18.75	6.25	0	0	0	[4]	0.983(0.028)
	Jiangsu	m17	67	11.94	17.91	1.49	20.9	14.93	0	2.99	10.45	4.48	1.49	2.99	2.99	1.49	0	5.97	0	[4]	0.996(0.004)
	Jiangxi	m18	23	0	34.78	0	26.07	21.74	4.35	4.35	8.7	0	0	0	0	0	0	0	0	[4]	0.996(0.014)
	Shanghai	m19	56	7.14	10.71	5.36	19.64	10.71	0	7.14	7.14	7.14	0	5.36	3.57	3.57	0	12.5	0	[4]	0.997(0.004)
	Sichuan	m20	70	4.29	22.86	1.43	18.57	11.43	4.29	11.43	11.43	1.43	1.43	0	2.86	4.29	1.43	2.86	0	[4]	0.996(0.003)
	Yunnan1	m21	43	4.65	20.93	4.65	13.95	20.93	0	4.65	18.6	0	0	0	6.98	2.33	0	0	2.33	[4]	0.992(0.008)
	Yunnan2	m22	59	5.08	16.95	5.08	18.64	15.25	6.78	3.39	16.95	1.69	1.69	3.39	0	0	1.69	3.39	0	[5]	0.989(0.007)
	Zhejiang	m23	61	4.92	24.59	8.2	8.2	11.48	6.56	16.39	3.28	3.28	3.28	1.64	6.56	0	0	1.64	0	[4]	0.996(0.004)
Southern minorities	Aini	m24	47	2.13	19.15	2.13	25.53	14.89	4.26	14.89	10.64	2.13	0	2.13	0	0	0	2.13	0	[8]	0.988(0.008)
	Bai1	m25	71	5.63	4.23	2.82	18.31	23.94	7.04	21.13	7.04	0	4.23	2.82	0	2.82	0	0	0	[8,9]	0.996(0.009)
	Bai2	m26	19	5.26	11.05	0	15.79	26.32	0	15.79	5.26	0	5.26	0	0	5.26	0	0	0	[8]	0.996(0.004)
	Bugan	m27	32	0	3.1	3.1	18.8	53.1	12.5	3.1	6.3	0	0	0	0	0	0	0	0	[10]	0.933(0.024)
	Buyang	m28	31	0	20.5	8.8	0	32.3	11.8	0	17.6	0	0	5.9	0	2.9	0	0	0	[10]	0.978(0.015)
	Blue Gelao	m29	30	3.3	13.3	13.3	16.7	16.6	0	13.3	20	0	0	3.3	0	0	0	0	0	[10]	0.956(0.019)
	Caolan	m30	30	0	23.3	0	6.7	13.3	3.3	10	20	0	0	13.3	3.3	6.6	0	0	0	[10]	0.993(0.011)
	Cun	m31	30	0	26.6	10	6.7	13.3	3.3	16.7	13.3	3.3	0	0	0	6.7	0	0	0	[10]	0.972(0.017)
	danga	m32	40	5	37.5	7.5	0	7.5	5	7.5	12.5	0	0	10	2.5	2.5	0	2.5	0	[10]	0.995(0.004)
	Dai	m33	118	5.4	17.9	8.9	10.7	14.3	0	12.5	14.3	0	0	0	1.8	10.8	0	3.6	0	[9-12]	0.996(0.004)
	Dornqdaye	m34	17	5.9	41.2	0	0	23.5	0	11.8	5.9	0	0	0	0	0	0	0	5.9	[10]	0.985(0.025)
	E	m35	33	0	31.2	0	9.4	28.2	0	0	6.2	0	0	3.1	9.4	9.4	0	3.1	0	[10]	0.952(0.026)
	hani	m36	33	12.12	18.18	3.03	27.27	21.21	0	12.12	0	0	0	0	0	3.03	3.03	0	0	[8]	0.996(0.009)
	hlai-qi	m37	34	0	32.3	0	2.9	8.8	8.8	23.5	11.8	2.9	0	2.9	2.9	2.9	0	0	0	[10]	0.989(0.010)
	Jiamao	m38	27	0	23	0	0	26.9	0	3.8	11.5	7.7	0	3.8	0	23	0	0	0	[10]	0.960(0.021)

	Jino	m39	18	5.56	11.11	5.56	0	16.67	0	27.78	16.67	0	5.56	0	11.11	0	0	0	0	[8]	0.974(0.029)
	Lachi	m40	30	0	32.4	8.8	0	5.8	2.9	11.8	26.5	0	0	0	0	8.8	0	0	2.9	[10]	0.970(0.017)
	Lahu1	m41	32	0	25	0	9.38	34.38	0	18.75	0	0	0	0	0	12.5	0	0	0	[11]	0.917(0.022)
	Lahu2	m42	15	0	20	6.67	26.67	33.33	0	0	6.67	6.67	0	0	0	0	0	0	0	[8]	1.000(0.024)
	Lahu3	m43	35	0	2.86	0	14.29	77.12	0	2.86	0	0	0	0	0	2.86	0	0	0	[12]	0.956(0.017)
	lingao	m44	31	6.7	13.2	6.7	3.3	29.9	6.7	6.7	10	6.7	0	0	3.3	3.3	0	0	3.3	[10]	0.996(0.009)
	Mak	m45	33	0	21.2	0	6	21.2	3	3	39.5	0	0	0	0	3	0	0	3	[10]	0.977(0.015)
	Maonan	m46	32	0	34.3	3.1	3.1	9.4	0	3.1	25	6.3	0	3.1	12.5	0	0	0	0	[10]	0.952(0.021)
	Miao-GX	m47	25	0	28	0	12	24	0	4	28	0	0	0	4	0	0	0	0	[13]	0.983(0.015)
	Miao-HN	m48	103	8.74	18.45	9.71	19.42	12.62	4.85	6.8	4.85	1.94	0	0.97	1.94	5.66	0.97	0.97	0	[13]	0.995(0.002)
	Miao-YN	m49	39	0	33.33	5.13	17.95	5.13	2.56	23.08	5.13	0	0	0	2.56	5.13	0	0	0	[13]	0.937(0.022)
	Mollao	m50	29	3.4	20.6	3.4	13.8	17.2	0	10.3	13.8	0	0	10.3	0	6.9	0	0	0	[10]	0.983(0.014)
	Mulam	m51	39	5.3	18.4	0	7.9	28.9	0	10.5	18.3	2.6	0	0	2.6	0	0	2.6	0	[10]	0.988(0.010)
	Naxi	m52	45	8.89	24.44	8.89	4.44	24.44	6.67	15.56	2.22	0	0	0	0	2.22	0	2.22	0	[8]	0.981(0.010)
	Nu	m53	30	30	10	0	20	16.67	3.33	3.33	0	0	0	0	0	0	0	16.67	0	[9]	0.857(0.038)
	Palyu	m54	30	3.3	0	23.3	13.3	6.7	0	0	33.3	0	0	13.3	3.3	3.3	0	0	0	[10]	0.938(0.024)
	Pou	m55	34	2.9	11.8	0	8.8	47	0	8.8	14.7	2.9	0	0	2.9	0	0	0	0	[10]	0.973(0.014)
	Pubiao	m56	25	0	12	0	8	16	8	16	28	0	0	0	0	4	0	8	0	[10]	0.977(0.018)
	Pumi	m57	36	14.29	2.86	22.86	17.14	8.57	8.57	20	0	0	0	0	2.86	0	0	2.86	0	[8]	0.980(0.016)
	Red Gelao	m58	31	0	2.8	0	2.8	11.1	44.4	0	11.1	2.8	0	0	22.2	0	0	0	2.8	[10]	0.892(0.040)
	Shui	m59	30	0	30	0	3.3	36.6	0	6.7	13.4	6.7	0	0	0	3.3	0	0	0	[10]	0.952(0.026)
	Then	m60	30	6.7	33.3	6.7	6.6	3.3	0	10	30	3.3	0	0	0	0	0	0	0	[10]	0.972(0.017)
	Tibetan	m61	99	25	21.43	1.79	5.36	7.14	5.36	10.71	35.71	0	0	8.93	1.79	1.79	0	0	0	[8,9,11]	0.989(0.002)
	Tujia	m62	96	0	13.33	3.33	16.67	43.33	6.67	3.33	6.67	0	3.33	0	6.67	0	0	0	0	[8]	0.993(0.003)
	Va	m63	58	0	2.78	0	25	44.44	0	19.44	0	0	8.33	0	0	0	0	0	0	[11,12]	0.972(0.010)
	Yao-GD	m64	35	2.86	42.86	0	5.71	20	0	8.57	5.71	2.86	0	0	5.71	5.71	0	0	0	[13]	0.975(0.013)
	Yao-HN	m65	24	4.17	37.5	0	20.83	12.5	0	16.67	0	0	0	0	8.33	0	0	0	0	[13]	0.995(0.016)
	Yao-YN	m66	77	1.3	40.26	5.19	18.18	3.9	2.6	10.39	5.19	0	0	0	2.6	9.09	0	1.3	0	[13]	0.976(0.007)
	yao-guangxi(IU)	m67	135	1.47	33.09	5.15	7.35	19.12	7.35	4.41	13.97	0	0	0	0.74	6.62	0	0.74	0	[13]	0.983(0.004)
	yao-guangxi(KIM)	m68	99	2.02	25.25	9.09	11.11	21.21	1.01	4.04	13.13	1.01	0	0	4.04	8.08	0	0	0	[13]	0.991(0.003)
	Yi	m69	56	10	17.5	2.5	15	15	5	20	12.5	2.5	0	0	0	0	0	0	0	[8]	0.995(0.004)
	Zhuang	m70	120	0	24.3	2.7	10.8	10.8	0	5.4	24.3	0	0	2.7	8.1	8.1	0	0	0	[9,10]	0.992(0.004)
Northern minorities	Daur	m71	45	2.2	15.6	6.7	24.4	0	8.8	0	20	0	0	0	2.2	4.4	2.2	8.9	4.4	[14]	0.979(0.009)
	Evenki	m72	47	4.3	10.6	19.1	31.9	2.1	8.5	0	0	0	0	0	0	0	0	4.3	19.1	[14]	0.956(0.011)
	Hui	m73	45	8.3	10.5	6.3	39.6	8.4	4.2	2.1	2.1	0	4.2	0	6.3	2.1	0	4.2	2.1	[15]	0.997(0.005)
	Kazakh	m74	53	3.8	3.8	13.2	13.2	7.6	5.7	3.8	1.9	1.9	1.9	0	1.9	0	0	11.3	30.3	[15]	0.995(0.005)
	Korean	m75	48	14.6	10.5	0	33.3	0	16.8	6.3	8.4	0	0	0	2.1	0	0	6.3	0	[14]	0.975(0.009)
	Mongolian	m76	48	8.2	2	10.2	20.4	18.3	16.3	4	2	0	2	0	2	0	0	0	14.3	[14,15]	0.993(0.003)
	Oroqen	m77	44	4.5	2.3	29.5	43.1	4.5	11.4	0	0	0	0	2.3	0	0	0	2.3	0	[14]	0.948(0.015)
	Uygur	m78	47	4.3	2.1	6.4	10.6	6.4	12.7	2.1	6.4	6.4	0	0	0	0	0	0	42.4	[15]	0.996(0.006)
	Uzbek	m79	58	3.4	5.1	1.7	22.4	8.5	6.8	3.4	0	0	0	3.4	3.4	1.7	1.7	1.7	34.2	[15]	0.996(0.004)

1. Keyser-Tracqui C, Crubezy E, Ludes B. Nuclear and mitochondrial DNA analysis of a 2,000-year-old necropolis in the Egyin Gol Valley of Mongolia. Am J Hum Genet. 2003; 73: 247-260.

2. Wang H, Ge B, Mair VH, Cai D, Xie C, Zhang Q, et al. Molecular genetic analysis of remains from Lamadong cemetery, Liaoning, China. Am J Phys Anthropol . 2007; 134: 404-411.

3. Changchun Y, Li X, Xiaolei Z, Hui Z, Hong Z. Genetic analysis on Tuoba Xianbei remains excavated from Qilang Mountain Cemetery in Qahar Right Wing Middle Banner of

Inner Mongolia. FEBS Lett. 2006; 580: 6242-6246.
4. Wen B, Li H, Lu D, Song X, Zhang F, He Y, et al. Genetic evidence supports demic diffusion of Han culture. Nature. 2004; 431: 302-305.
5. Yao YG, Kong QP, Bandelt HJ, Kivisild T, Zhang YP. Phylogeographic differentiation of mitochondrial DNA in Han Chinese. Am J Hum Genet. 2002; 70: 635-651.
6. Yao YG, Kong QP, Man XY, Bandelt HJ, Zhang YP. Reconstructing the evolutionary history of China: a caveat about inferences drawn from ancient DNA. Mol Biol Evol. 2003; 20: 214-219.
7. Kivisild T, Tolk HV, Parik J, Wang Y, Papiha SS, Bandelt HJ, et al. The emerging limbs and twigs of the East Asian mtDNA tree. Mol Biol Evol. 2002; 19: 1737-1751.
8. Wen B, Xie X, Gao S, Li H, Shi H, Song X, et al. Analyses of genetic structure of Tibeto-Burman populations reveals sex-biased admixture in southern Tibeto-Burmans. Am J Hum Genet. 2004; 74: 856-865.
9. Yao YG, Nie L, Harpending H, Fu YX, Yuan ZG, Zhang YP. Genetic relationship of Chinese ethnic populations revealed by mtDNAsequence diversity. Am J Phys Anthropol. 2002; 118: 63-76.
10. Li H, Cai X, Winograd-Cort ER, Wen B, Cheng X, QinZ, et al. Mitochondrial DNA diversity and population differentiation in southern East Asia. Am J Phys Anthropol. 2007; 134: 481-488.
11. Qian YP, Chu ZT, Dai Q, Wei CD, Chu JY, TajimaA, et al. Mitochondrial DNA polymorphisms in Yunnan nationalities in China. J Hum Genet. 2001; 46: 211-220.
12. Yao YG, Zhang YP. Phylogeographic analysis of mtDNA variation in four ethnic populations from Yunnan Province: new data and a reappraisal. J Hum Genet. 2002; 47: 311-318.
13. Wen B, Li H, Gao S, Mao X, Gao Y, LiF, et al. Genetic structure of Hmong-Mien speaking populations in East Asia as revealed by mtDNA lineages. Mol Biol Evol. 2005; 22: 725-734.
14. Kong QP, Yao YG, Liu M, Shen SP, Chen C, ZhuCL, et al. Mitochondrial DNA sequence polymorphisms of five ethnic populations from northern China. Hum Genet. 2003; 113: 391-405.
15. Yao YG, Kong QP, Wang CY, Zhu CL, Zhang YP. Different matrilineal contributions to genetic structure of ethnic groups in the silk road region in china. Mol Biol Evol. 2004; 21: 2265-2280.
