# Supplementary material for: Ancient DNA Reveals That the Genetic Structure of the Northern Han Chinese Was Shaped Prior to 3,000 Years Ago
Source: PLoS One. 2015 May 4;10(5):e0125676. doi: 10.1371/journal.pone.0125676 (PMC4418768; doi:10.1371/journal.pone.0125676)
Supplement: S4 Table — (PDF) [file pone.0125676.s008.pdf]

Table S4 Estimated percentages of Y chromosome haplogroups shared among HB and modern Chinese populations.

Group	Population	code	size	Estimated percentage of Y chromosome Haplogroup (%) D	C	F	G	I	J	K	N	N1	O	O1	O2	O3	P	Q	Q1a1	R	Reference
Ancient people	Hengbei	HB	27	0	0	0	0	0	0	0	0	3.71	14.81	0	7.41	33.33	0	0	40.74	0	[1]
Northern Han	Hebei	y1	14	0	0	14.29	0	0	0	7.14	0	0	0	7.14	0	71.43	0	0	0	0	[2]
	Henan	y2	50	0	4	4	0	0	0	22	0	0	0	8	0	52	2	0	8	0	[2]
	Gansu	y3	60	8.33	11.67	10	0	0	0	16.67	0	0	0	8.33	1.67	36.67	1.67	0	5	0	[2]
	Liaoning	y4	48	2.08	2.08	22.92	0	0	0	16.67	0	0	0	4.17	2.08	45.83	0	0	4.17	0	[2]
	Neimeng	y5	60	5	20	6.67	0	0	0	13.33	0	0	0	1.67	1.67	48.34	0	0	3.33	0	[2]
	Shandong1	y6	85	3.53	16.47	3.53	0	0	0	14.12	0	0	0	0	1.18	56.46	0	0	4.71	0	[2]
	Shandong2	y7	100	0	4	11	0	0	0	13	0	0	0	6	1	62	0	0	3	0	[2]
	Shannxi1	y8	63	4.76	3.17	6.35	0	0	0	17.46	0	0	0	1.59	1.59	60.32	3.17	0	1.59	0	[2]
	Shannxi2	y9	27	0	0	11.11	0	0	0	33.33	0	0	0	3.7	0	38.15	0	0	3.7	0	[2]
	Xinjiang	y10	51	1.96	3.92	5.88	0	0	0	17.65	0	0	0	3.92	0	58.82	3.92	0	3.92	0	[2]
	nHan1	y11	82	0	8.54	2.44	0	0	0	21.95	0	0	0	9.76	0	52.44	0	0	4.88	0	[3]
	nHan2	y12	44	0	4.55	0	2.27	0	2.27	0	2.27	6.82	0	0	6.82	65.91	0	4.55	0	2.73	[4]
	nHan3	y13	44	0	4.55	0	2.27	0	2.27	11.36	0	0	0	0	6.82	65.91	4.55	0	0	2.27	[5]
Southern Han	Anhui	y14	22	0	13.6	0	0	0	0	18.18	0	0	0	18.18	0	35.45	0	0	4.55	0	[2]
	Fujian	y15	148	0.68	2.7	2.03	0	0	0	14.19	0	0	0	2.71	2.7	74.33	0	0	0.68	0	[2]
	Guangdong	y16	64	1.56	4.69	0	0	0	0	12.5	0	0	0	7.81	18.75	53.13	0	0	1.56	0	[2]
	Guangxi	y17	26	0	7.69	0	0	0	0	15.38	0	0	0	15.39	26.89	34.61	0	0	0	0	[2]
	Hubei	y18	18	0	5.56	0	0	0	0	11.11	0	0	0	16.67	0	66.67	0	0	0	0	[2]
	Hunan	y19	15	0	0	0	0	0	0	13.33	0	0	0	13.33	13.33	60	0	0	0	0	[2]
	Jiangsu	y20	100	2	8	3	0	0	0	19	0	0	0	18	4	46	0	0	2	0	[2]
	Jiangxi	y21	21	4.76	4.76	9.52	0	0	0	19.05	0	0	0	14.29	4.76	42.86	0	0	0	0	[2]
	Shanghai	y22	55	3.64	7.27	0	0	0	0	16.36	0	0	0	25.46	0	42.93	0	0	3.64	0	[2]
	Sichuan	y23	63	1.59	4.76	0	0	0	0	15.87	0	0	0	7.94	12.69	57.15	0	0	0	0	[2]
	Yunnan1	y24	27	0	11.1	3.7	0	0	0	3.7	0	0	0	3.7	3.7	74.07	0	0	0	0	[2]
	Yunnan2	y25	66	3.03	6.06	3.03	0	0	0	22.73	0	0	0	0	3.03	59.1	0	0	3.03	0	[2]
	Zhejiang	y26	106	0	9.43	0	0	0	0	5.66	0	0	0	27.36	4.72	50.95	0	0	1.89	0	[2]
	sHan1	y27	280	0.36	7.86	1.43	0	0	0	12.86	0	0	0	16.79	4.29	55	0	0	1.43	0	[3]
	sHan2	y28	40	0	5	0	0	0	0	0	2.5	15	0	15	30	32.5	0	0	0	0	[4]
	sHan3	y29	40	0	5	0	0	0	0	0	17.5	0	0	0	15	30	32.5	0	0	0	[5]
Southern minorities	beizhuang	y30	22	0	0	0	0	0	0	0	0	0	13.64	4.55	72.73	9.1	0	0	0	0	[6]
	blue gelao	y31	30	0	0	0	0	0	0	0	3.33	0	0	13.33	60	16.67	6.67	0	0	0	[6]
	bolyu	y32	30	3.33	0	0	0	0	0	3.33	0	0	10	13.33	23.33	46.67	0	0	0	0	[6]
	buyang	y33	32	3.13	0	6.25	0	0	0	6.25	0	0	9.38	3.13	71.88	0	0	0	0	0	[6]
	caomiao	y34	33	0	0	0	0	0	0	9.09	0	0	0	9.09	3.03	78.79	0	0	0	0	[6]
	dong	y35	38	0	21.05	0	0	0	0	5.26	0	0	10.53	0	39.47	13.69	0	0	0	0	[6]
	E	y36	31	0	3.23	3.23	0	0	0	9.68	0	0	16.13	6.45	54.84	6.45	0	0	0	0	[6]
	kucun	y37	21	19.05	0	52.38	0	0	0	0	0	0	0	0	0	28.57	0	0	0	0	[7]
	jiamao	y38	27	0	0	0	0	0	0	0	0	0	25.93	51.85	22.22	0	0	0	0	0	[6]
	laqua	y39	25	0	0	0	0	0	0	0	0	0	32	4	60	4	0	0	0	0	[6]
	li	y40	11	0	0	0	0	0	0	0	0	0	0	27.27	63.64	9.09	0	0	0	0	[3]
	man-caolan	y41	30	0	10	0	0	0	0	10	0	0	53.33	3.33	20	3.33	0	0	0	0	[6]
	maonan	y42	32	0	9.38	9.38	0	0	0	15.63	0	0	0	0	56.25	9.38	0	0	0	0	[6]
	mollao	y43	30	0	10	0	0	0	0	3.33	0	0	13.33	3.33	63.33	3.33	0	0	0	0	[6]
	mosuo	y44	47	27.65	6.38	2.13	0	0	0	31.91	0	0	0	8.51	0	23.4	0	0	0	0	[8]

	mulam	y45	40	12.5	2.5	7.5	0	0	0	5	0	0	0	30	30	12.5	0	0	0	0	[6]
	nanzhuang	y46	15	0	0	0	0	0	0	0	0	0	13.33	86.67	0	0	0	0	0	0	[6]
	naxi	y47	40	37.5	2.5	0	0	0	0	10	0	0	0	0	47.5	2.5	0	0	0	0	[8]
	pumi	y48	47	72.33	2.13	4.26	0	0	0	6.38	0	0	0	4.26	0	8.51	2.13	0	0	0	[8]
	red gelao	y49	31	0	0	3.23	0	0	0	0	6.45	0	0	22.58	22.58	16.13	29	0	0	0	[6]
	she	y50	11	0	18.18	0	0	0	0	9.09	0	0	0	0	9.09	63.63	0	0	0	0	[3]
	shui	y51	50	0	0	8	0	0	0	10	0	0	0	18	44	20	0	0	0	0	[6]
	tujia	y52	49	2.04	18.37	0	0	0	0	0	0	4.08	0	8.16	4.08	53.06	0	0	0	4.08	[9]
	white gelao	y53	14	0	0	0	0	0	0	0	0	0	0	35.71	14.29	42.86	7.14	0	0	0	[6]
	yangyueb	y54	74	6.76	2.7	0	0	0	0	0	0	0	0	51.39	8.11	31.08	0	0	0	0	[6]
	yangyuen	y55	51	1.96	5.88	0	0	0	0	1.96	0	0	31.37	29.41	1.96	26.45	0	0	0	0	[6]
	Yao1	y56	60	1.67	20	0	0	0	0	0	0	0	0	1.67	1.67	53.33	0	0	0	0	[9]
	Yao2	y57	10	0	50	0	0	0	0	20	0	0	0	0	0	30	0	0	0	0	[3]
	Yao3	y58	10	30	20	0	0	0	0	0	0	0	0	0	40	10	0	0	0	0	[3]
	Yao4	y59	14	57.1	0	7.14	0	0	0	0	0	0	7.14	0	0	28.57	0	0	0	0	[7]
	Yao5	y60	60	1.67	20	0	0	0	0	0	0	0	0	1.67	23.33	48.33	0	0	0	0	[4]
	yerong	y61	16	0	0	0	0	0	0	0	0	0	0	0	68.75	31.25	0	0	0	0	[6]
	yi	y62	14	14.29	0	0	0	0	0	42.86	0	0	0	0	14.29	28.55	0	0	0	0	[3]
	Yi2	y63	50	12	8	38	0	0	0	18	0	0	0	2	4	20	0	0	0	0	[8]
	Yi3	y64	43	16.3	2.33	4.7	0	0	0	0	2.33	30.2	0	0	11.65	32.56	0	0	0	0	[4]
	Yi4	y65	43	16.28	2.33	4.65	0	0	0	32.56	0	0	0	0	11.63	32.56	0	0	0	0	[5]
	Zang	y66	75	49.3	2.67	1.3	0	0	1.33	4	0	0	1.33	0	0	33.33	0	0	0	6.67	[5]
	Zhuang1	y67	28	3.57	3.57	7.14	0	0	0	3.57	0	0	0	17.86	25	39.28	0	0	0	0	[3]
	Zhuang2	y68	63	11.1	12.7	0	0	0	0	0	0	6.35	4.76	19	23.76	22.22	0	0	0	0	[10]
	Zhuang3	y69	20	0	0	0	0	0	0	0	0	0	5	10	70	15	0	0	0	0	[4]
Northern minorities	Baoan	y70	27	14.8	0	0	0	0	0	22.22	3.7	0	7.41	0	18.5	14.81	0	0	0	18.52	[11]
	buryat	y71	81	0	60.49	0	1.23	0	1.24	0	0	30.86	0	0	0	2.47	0	0	0	3.7	[4]
	Evenk	y72	95	0	68.42	0	0	5.26	2.11	0	0	18.95	0	0	0	0	0	4.21	0	1.05	[4]
	Evenk2	Y73	31	0	74.2	0	0	3.23	0	0	0	12.9	0	0	0	0	0	3.23	0	6.45	[4]
	evenki	y74	41	0	43.9	0	0	0	0	4.88	0	0	0	2.44	7.32	34.12	0	0	0	4.88	[5]
	hui	y75	22	0	22.73	22.73	0	0	0	13.64	0	0	9.09	0	0	13.64	0	0	0	18.2	[7]
	Kazak	y76	41	2.44	58.5	4.88	2.44	0	2.44	2.44	9.76	0	0	0	0	7.32	0	0	0	9.76	[10]
	kirkiz	y77	45	0	8.89	0	0	0	0	2.22	4.44	0	4.44	0	0	4.44	2.22	2.22	0	0	[10]
	Manchu1	y78	41	0	26.9	0	0	0	1.92	0	0	5.77	5.77	3.85	9.62	38.46	0	0	0	7.69	[4]
	Manchu2	y79	41	0	43.91	0	0	0	0	0	0	4.88	0	2.44	24.88	24.39	0	9.76	0	4.88	[4]
	Manchu3	y80	52	0	26.92	0	0	0	1.92	5.77	0	0	5.77	3.85	5.77	38.46	0	0	0	7.68	[4]
	Mongolia1	y81	24	4.17	58.33	8.33	0	0	0	12.5	0	0	0	4.17	0	8.33	0	0	0	4.17	[3]
	mongolia2	y82	50	4	40	0	0	0	0	4	6	0	4	0	2	32	0	0	2	6	[10]
	Oroqen1	y83	22	0	90.91	0	0	0	0	0	0	4.55	0	0	4.55	0	0	0	0	0	[4]
	Ororqen2	y84	23	0	91.3	0	0	0	0	0	0	4.35	0	0	4.35	0	0	0	0	0	[5]
	sala1	y85	32	6.25	6.25	938	0	0	0	0	0	0	21.88	0	0	31.25	3.13	0	0	21.9	[7]
	sala2	y86	35	0	0	0	0	0	0	0	11.43	0	8.57	2.86	2.86	20	0	0	0	54.3	[10]
	Tu	y87	50	8	28	2	0	0	2	2	10	0	6	6	2	26	0	2	0	6	[10]
	Tatal	y88	33	0	0	0	0	33.33	0	0	0	0	0	0	0	6.06	0	0	0	60.6	[10]
	Xibe	y89	32	0	12.5	0	0	0	3.13	9.38	15.63	0	21.88	12.5	0	21.88	0	0	0	3.13	[10]
	Yugu	y90	32	43.8	3.13	6.25	0	0	6.25	6.25	6.25	0	3.13	0	0	15.63	0	0	0	9.38	[10]

1.Zhao YB, Zhang Y, Li HJ, Cui YQ, Zhu H, Zhou H. Ancient DNA evidence reveals that the Y chromosome haplogroup Q1a1 admixed into the Han Chinese 3,000 years ago. Am J Hum Biol. 2014; 26: 813-821.

2. Wen B, Li H, Lu D, Song X, Zhang F, HeY, et al. Genetic evidence supports demic diffusion of Han culture. Nature. 2004; 431: 302-305.
3. Su B, Xiao J, Underhill P, Deka R, Zhang W, AkeyJ, et al. Y-Chromosome evidence for a northward migration of modern humans into Eastern Asia during the last Ice Age. Am J Hum Genet. 1999; 65: 1718-1724.
4. Hammer MF, Karafet TM, Park H, Omoto K, Harihara S, StonekingM, et al. Dual origins of the Japanese: common ground for hunter-gatherer and farmer Y chromosomes. J Hum Genet. 2006; 51: 47-58.
5. Karafet T, Xu L, Du R, Wang W, Feng S, WellsRS, et al. Paternal population history of East Asia: sources, patterns, and microevolutionary processes. Am J Hum Genet. 2001; 69: 615-628.
6. Li H, Wen B, Chen S, Su B, Pramoonjago P, LiuY, et al. Paternal genetic affinity between Western Austronesians and Daic populations. BMC Evol Biol. 2008; 15: 146.
7. Black ML, Wise CA, Wang W, Bittles AH. Combining genetics and population history in the study of ethnic diversity in the People's Republic of China. Hum Biol. 2006; 78: 277-293.
8. Wen B, Shi H, Ren L, Xi HF, Li KY, ZhangWY, et al. Study on the Y chromosome, mitochondrial DNA polymorphism and the ethnic origin of the Mosuo people in Ninglang, Yunnan province.Science in China(SERIES C). 2003; 33: 375-384.
9. Karafet TM, Hallmark B, Cox MP, Sudoyo H, Downey S, LansingJS, et al. Major east-west division underlies Y chromosome stratification across Indonesia. Mol Biol Evol. 2010; 27: 1833-1844.
10. Zhao Q, Pan SL, Qin ZD, Cai XY, Lu Y, XuJS, et al. The Minz Zhuang of Napo County, Duangxi: Legacy of the Thai-Lao Migration South. Communication on Contemporary Anthropology. 2010; 16: 22-26.
11. Shou WH, Qiao EF, Wei CY, Dong YL, Tan SJ, ShiH, et al. Y-chromosome distributions among populations in Northwest China identify significant contribution from Central Asian pastoralists and lesser influence of western Eurasians. J Hum Genet. 2010; 55: 314-322.
