# Supplementary material for: Ancient DNA Reveals That the Genetic Structure of the Northern Han Chinese Was Shaped Prior to 3,000 Years Ago
Source: PLoS One. 2015 May 4;10(5):e0125676. doi: 10.1371/journal.pone.0125676 (PMC4418768; doi:10.1371/journal.pone.0125676)
Supplement: S5 Table — (PDF) [file pone.0125676.s009.pdf]

Table S5 Population pairwise Fst values and Fst p values between ancient and current populations.

Group	Population	HB		XN		XB	
		F_{ST}	P value	F_{ST}	P value	F_{ST}	P value
NH	Gansu	0.01922	0.02703±0.0194	0.01751	0.01802±0.0182	0.01977	0.00901±0.0091
	Liaoning	-0.00222	0.67568±0.0530	0.02091	0.00000±0.0000	0.01570	0.00901±0.0091
	Neimeng	-0.00038	0.51351±0.0451	0.02581	0.00000±0.0000	0.01464	0.02703±0.0194
	Qinghai	0.00173	0.32432±0.0492	-0.00061	0.49550±0.0434	0.00789	0.12613±0.0278
	Shandong	-0.00115	0.49550±0.0412	0.02205	0.00000±0.0000	0.02219	0.00000±0.0000
	Shannxi	-0.00062	0.45946±0.0697	0.00711	0.19820±0.0402	0.01353	0.04505±0.0203
	Xinjiang	-0.00468	0.71171±0.0567	0.01149	0.07207±0.0227	0.01033	0.03604±0.0148
SH	Anhui	0.01437	0.03604±0.0201	0.04602	0.00000±0.0000	0.03318	0.00000±0.0000
	Fujian	0.01038	0.06306±0.0237	0.05158	0.00000±0.0000	0.03286	0.00000±0.0000
	Guangdong	0.03647	0.00000±0.0000	0.08323	0.00000±0.0000	0.06393	0.00000±0.0000
	Guangxi	0.04979	0.00000±0.0000	0.10557	0.00000±0.0000	0.06929	0.00000±0.0000
	Hubei	0.01843	0.02703±0.0139	0.05960	0.00000±0.0000	0.04157	0.00000±0.0000
	Hunan	0.02605	0.01802±0.0121	0.05931	0.00901±0.0091	0.03330	0.02703±0.0139
	Jiangsu	-0.00024	0.38739±0.0622	0.02667	0.00000±0.0000	0.02014	0.01802±0.0121
	Jiangxi	0.05291	0.00000±0.0000	0.11513	0.00000±0.0000	0.09198	0.00000±0.0000
	Shanghai	0.01002	0.04505±0.0203	0.03422	0.00000±0.0000	0.01554	0.01802±0.0121
	Sichuan	0.00227	0.25225±0.0521	0.03292	0.00000±0.0000	0.03022	0.00000±0.0000
	Yunnan	0.01551	0.00901±0.0091	0.05292	0.00000±0.0000	0.03764	0.00000±0.0000
	Zhejiang	0.00667	0.15315±0.0273	0.03203	0.00000±0.0000	0.02551	0.00000±0.0000
NM	Daur	0.00622	0.10811±0.0264	0.03099	0.00000±0.0000	0.02354	0.00901±0.0091
	Evenki	0.01642	0.02703±0.0139	0.02066	0.00901±0.0091	0.01427	0.04505±0.0203
	Kazak	0.01364	0.03604±0.0201	0.03535	0.00901±0.0091	0.02358	0.00000±0.0000
	Korean	0.01564	0.02703±0.0139	0.01911	0.00901±0.0091	0.01778	0.00901±0.0091
	Mongolian	0.00210	0.36036±0.0507	0.01764	0.00000±0.0000	0.01340	0.05405±0.0278
	Oroqen	0.03465	0.00000±0.0000	0.02431	0.00901±0.0091	0.00902	0.13514±0.0311
	Tu	-0.00392	0.63063±0.0304	0.01335	0.09009±0.0271	0.00761	0.15315±0.0194
	Uygur	0.00880	0.08108±0.0286	0.02524	0.00000±0.0000	0.02140	0.02703±0.0139
	Uzbek	0.01355	0.04505±0.0244	0.03288	0.00000±0.0000	0.03502	0.00000±0.0000
SM	Aini	0.00857	0.07207±0.0264	0.04084	0.00000±0.0000	0.02632	0.00901±0.0091
	Bai	0.00961	0.04505±0.0203	0.03365	0.00000±0.0000	0.03091	0.00000±0.0000
	Hani	0.00718	0.17117±0.0252	0.04081	0.00901±0.0091	0.03444	0.00000±0.0000
	Jino	0.00552	0.31532±0.0455	0.04735	0.00901±0.0091	0.02991	0.01802±0.0121
	Lahu	0.07061	0.00000±0.0000	0.13173	0.00000±0.0000	0.09835	0.00000±0.0000
	Lisu	0.03123	0.00000±0.0000	0.02732	0.00901±0.0091	0.03144	0.00901±0.0091
	Naxi	0.02078	0.01802±0.0121	0.05167	0.00000±0.0000	0.03836	0.00000±0.0000
	Nu	0.05657	0.00000±0.0000	0.04613	0.00901±0.0091	0.06564	0.00000±0.0000
	Pumi	0.04963	0.00000±0.0000	0.03337	0.00901±0.0091	0.03131	0.00901±0.0091
	Tujia	0.00092	0.29730±0.0471	0.03097	0.00000±0.0000	0.02081	0.00000±0.0000
	Yi	0.00681	0.12613±0.0242	0.03003	0.00000±0.0000	0.02722	0.00000±0.0000
	Miao	0.00806	0.05405±0.0242	0.03522	0.00000±0.0000	0.02637	0.00000±0.0000
	Yao	0.03929	0.00000±0.0000	0.08255	0.00000±0.0000	0.06818	0.00000±0.0000
	Blue Gelao	0.02934	0.00000±0.0000	0.05873	0.00000±0.0000	0.03229	0.00000±0.0000
	Caolan	0.04699	0.00000±0.0000	0.09356	0.00000±0.0000	0.06859	0.00000±0.0000
	Dai	0.01539	0.00901±0.0091	0.04553	0.00000±0.0000	0.02515	0.00000±0.0000
	Dornqdaye	0.05250	0.00901±0.0091	0.11139	0.00000±0.0000	0.07931	0.00000±0.0000
	E	0.06851	0.00000±0.0000	0.11166	0.00000±0.0000	0.08665	0.00000±0.0000
	Lachi	0.05876	0.00000±0.0000	0.10886	0.00000±0.0000	0.07164	0.00000±0.0000
	Mak	0.05457	0.00000±0.0000	0.09870	0.00000±0.0000	0.07168	0.00000±0.0000
	Maonan	0.08064	0.00000±0.0000	0.12676	0.00000±0.0000	0.09178	0.00000±0.0000
	Mollao	0.04818	0.00000±0.0000	0.06382	0.00000±0.0000	0.05101	0.00000±0.0000
	Mulam	0.03586	0.00000±0.0000	0.06232	0.00000±0.0000	0.05037	0.00000±0.0000
	Pou	0.03730	0.00000±0.0000	0.07015	0.00000±0.0000	0.04932	0.00000±0.0000
	Pubiao	0.03915	0.00000±0.0000	0.06120	0.00000±0.0000	0.03724	0.00000±0.0000
	Shui	0.06477	0.00000±0.0000	0.11521	0.00000±0.0000	0.08295	0.00000±0.0000
	Then	0.06647	0.00000±0.0000	0.10629	0.00000±0.0000	0.07239	0.00000±0.0000
	Zhuang	0.04322	0.00000±0.0000	0.08678	0.00000±0.0000	0.06532	0.00000±0.0000
	Bugan	0.07337	0.00000±0.0000	0.10739	0.00000±0.0000	0.07289	0.00000±0.0000
	Palyu	0.06155	0.00000±0.0000	0.09586	0.00000±0.0000	0.05616	0.00000±0.0000
	Va	0.09249	0.00000±0.0000	0.14204	0.00000±0.0000	0.10669	0.00000±0.0000

HB, ancient Hengbei people; XN, Xiongnu; XB, Xianbei; NH, northern Han; SH, southern Han; NM, northern Minorities; SM, southern Minorities.
